# Supplementary material for: Refractive index as an indicator for dynamic protein condensation in cell nuclei
Source: Biophys Rep (N Y). 2025 Nov 1;5(4):100235. doi: 10.1016/j.bpr.2025.100235 (PMC12666831; doi:10.1016/j.bpr.2025.100235)
Supplement: Document S1. Figures S1–S5 and Table S1 [file mmc1.pdf]

Biophysical Reports, Volume *5*

Supplemental information

Refractive index as an indicator for dynamic protein condensation in cell nuclei

Orlando Marin, Peter Kirchweger, Arina Dalaloyan, Yoav Barak, and Michael Elbaum

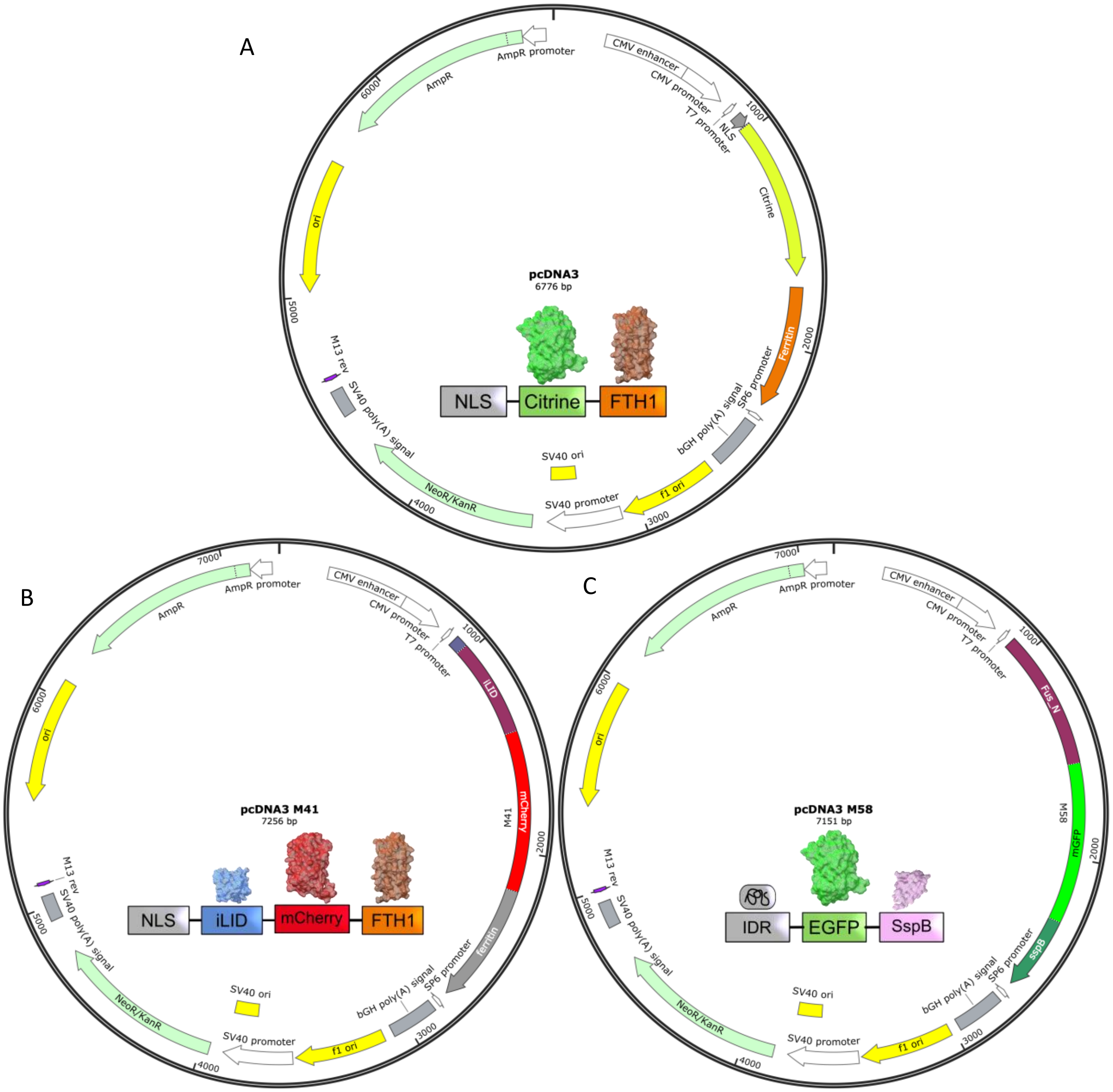

Figure S1: Plasmid maps of (A) SMPA, (B) the first and (C) second part of the Correlet system in the pcDNA3 vector: (A) NLS-Citrine-ferritin, (B) NLS-iLid-mCherry-Ferritin, and (C) IDR region of FUS_N-EGFP-SspB.

PBD IDs used in Fig. 1: **GFP**: 1GFL: https://doi.org/10.2210/pdb1GFL/pdb; **24-mer human ferritin**: 5N27: https://doi.org/10.2210/pdb5N27/pdb; **iLID domain**: 4WF0: https://doi.org/10.2210/pdb4WF0/pdb; **mCHerry**: 6YLM: https://doi.org/10.2210/pdb6YLM/pdb; **SspB**: 1OX9: https://doi.org/10.2210/pdb1OX9/pdb.

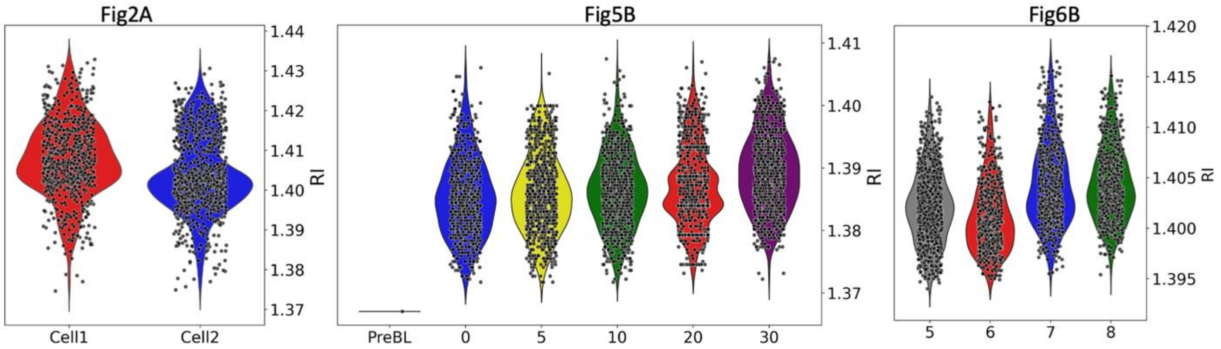

Figure S2: Violin plots showing the individual data points used to produce the plots. The left, middle and right panel show the plots from Fig 2A, Fig5B, and from Fig6B, respectively.

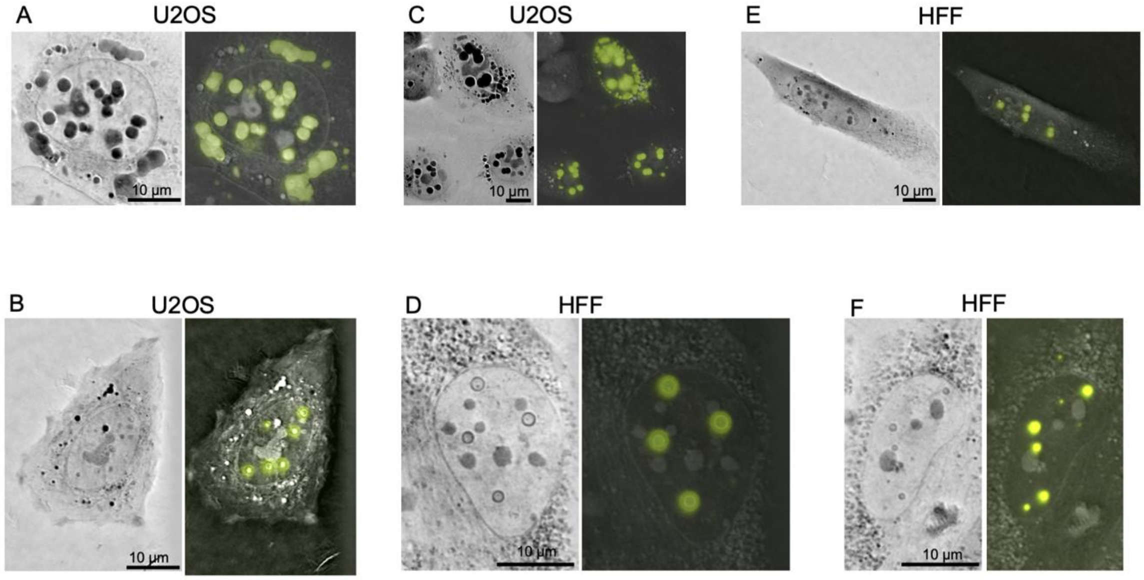

Figure S3. Additional Examples of SMPA in U2OS and HFF cells. SMPA can be reproduced in U2OS (A,B,C) and HFF cells (D,E,F). The thin spherical shells are seen in both cell types (B, D, F). Scale bars are 10 µm.

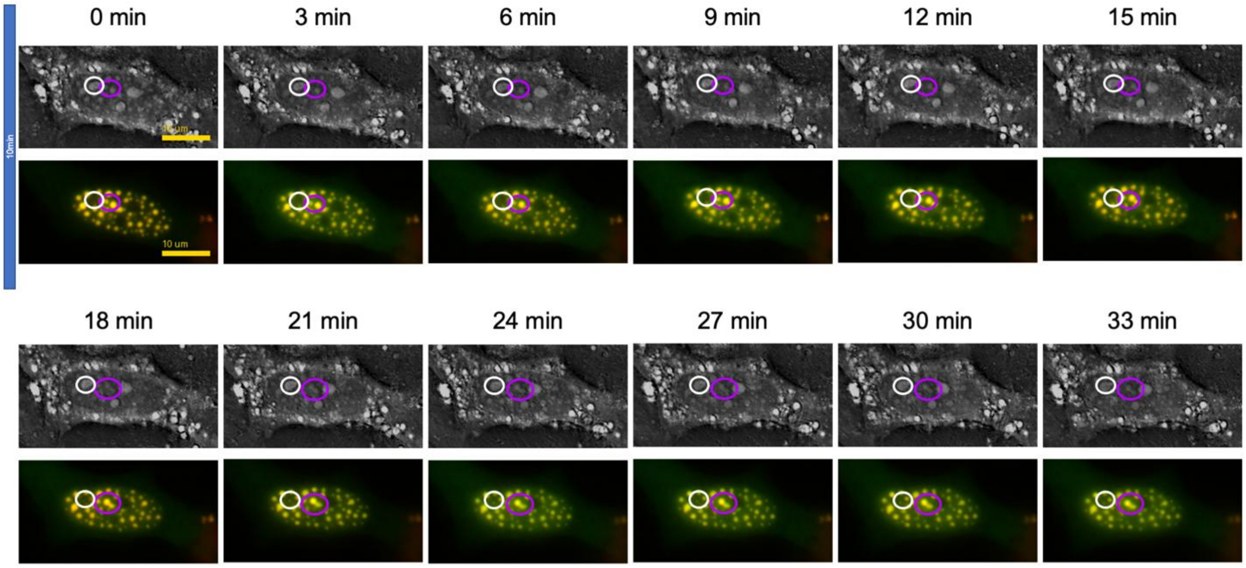

Figure S4: Long observation of Corelets. After 10 min blue light exposure, the same cell was imaged for 33 min (1x per minute); an image every 3 min is depicted. The purple outline indicates a persistent Corelet, and the white outline is a non-fluorescent nucleolus.

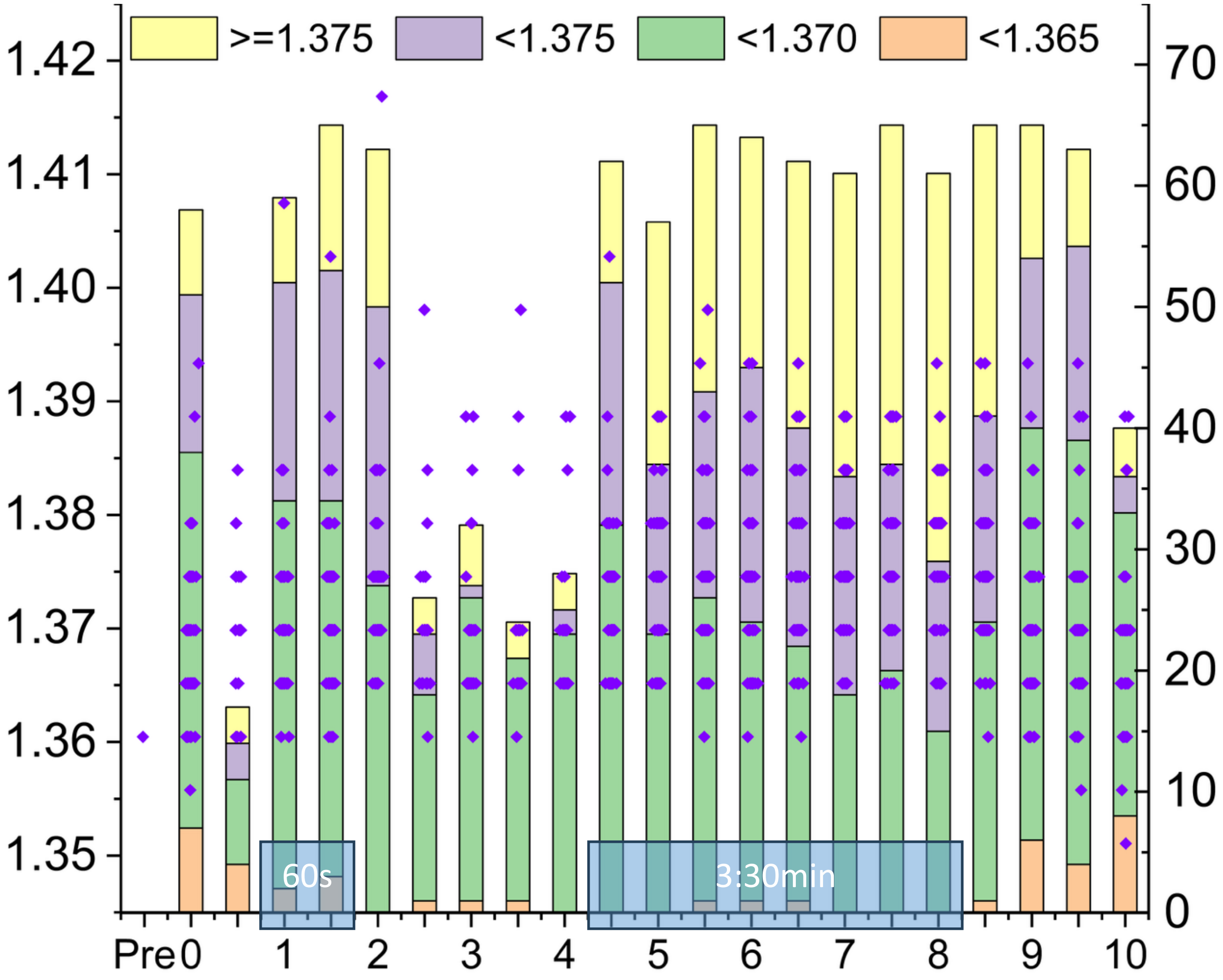

Figure S5: Quantification of Maximum values from Fig8. Plotted are rounded MAX values of the ROIs extracted from the timeseries shown in Fig8. Purple shows the actual datapoints, and counts appear on the right side vertical axis. Orange indicates MAX values below 1.365, green between 1.365 and 1.37, purple between 1.37 and 1.375, and yellow shows values above 1.375. The number of median and maximum values in each category are shown in Table S1.

TIME	TOTAL DATAPOINTS		<1.365		<1.370		<1.375		>=1.375	
	MED	MAX	MED	MAX	MED	MAX	MED	MAX	MED	MAX
**0**	54	58	33	7	21	31	0	13	0	7
**0.5**	15	17	9	4	5	7	1	3	0	3
**1**	54	59	27	2	26	32	1	18	0	7
**1.5**	54	65	25	3	26	31	3	19	0	12
**2**	58	63	26	0	30	27	1	23	1	13
**2.5**	25	26	14	1	11	17	0	5	0	3
**3**	29	32	19	1	10	25	0	1	0	5
**3.5**	24	24	14	1	10	20	0	0	0	3
**4**	29	28	17	0	11	23	1	2	0	3
**4.5**	57	62	25	0	31	32	0	20	1	10
**5**	56	57	28	0	28	23	0	14	0	20
**5.5**	60	65	34	1	26	25	0	17	0	22
**6**	64	64	32	1	32	23	0	21	0	19
**6.5**	62	62	30	1	32	21	0	18	0	22
**7**	59	61	27	0	32	18	0	18	0	25
**7.5**	60	65	23	0	35	20	2	17	0	28
**8**	58	61	25	0	33	15	0	14	0	32
**8.5**	63	65	23	1	38	23	2	17	0	24
**9**	56	65	28	6	27	34	1	14	0	11
**9.5**	56	63	28	4	27	35	1	16	0	8
**10**	32	40	21	8	11	25	0	3	0	4

Table S1: Analysis of Fig 8. Number of Datapoints in the categories below 1.365, between 1.365 and 1.37, between 1.37 and 1.375, and greater than 1.375.

Movie captions:

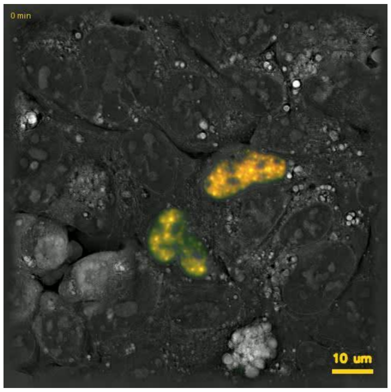

Movie S1: Image compilation the time series depicted in Figure 4. The entire field of view is shown as an overlay of the three channels. Scale bar 10 µm. Time stamps appear at the upper left corner.

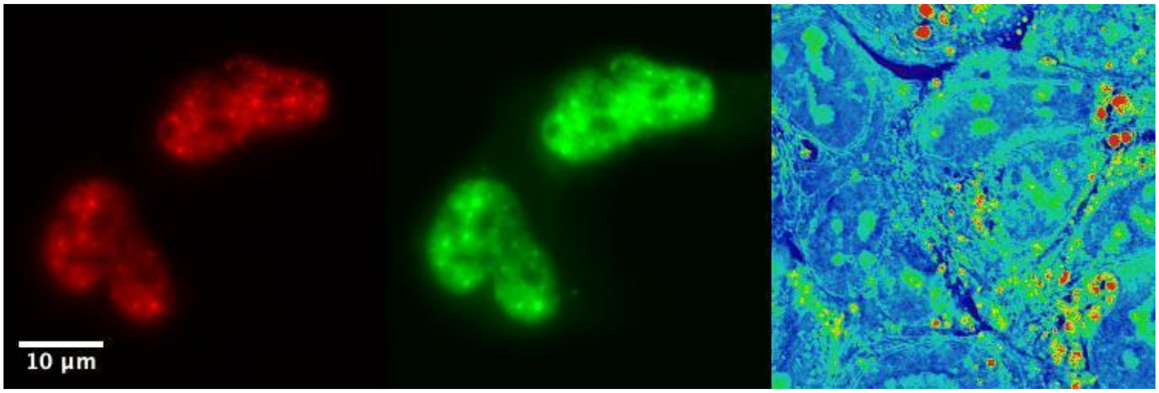

Movie S2: Image compilation of the Ft-mCherry, FUS-GFP and RI channels of the time series depicted in Figure 4. Blue frames indicate the timing of the photo-activation. The same area is shown as in Fig 4. Scale bar 10 µm.

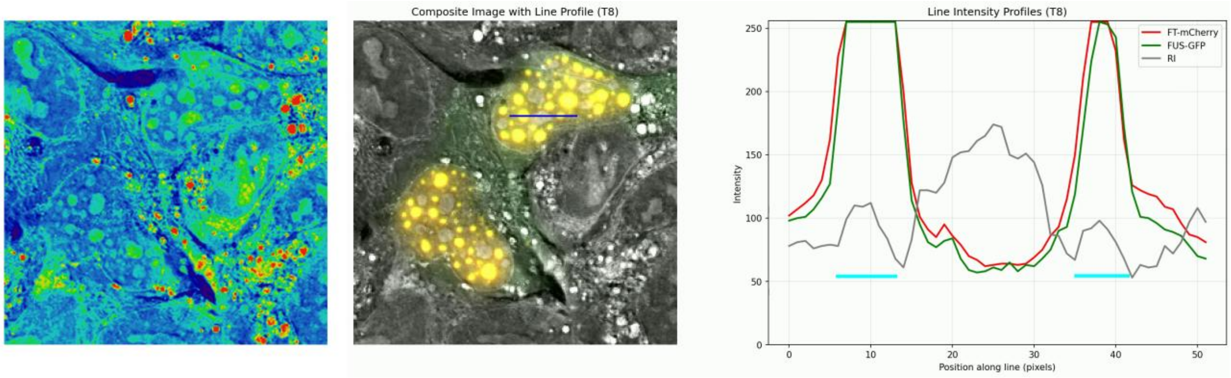

Movie S3: A line profile across the time series of Figure 4. The panel on the left repeats the frames in Fig 4. The center panel shows a composite image with combined fluorescence overlaid. The frame on the right shows intensity traces across the line shown in blue in the center panel. The green, red and black lines represent the FUS-GFP, Ft-mCherry and RI channels, respectively. The elevated RI between the two fluorescence peaks is a nucleolus. Note the dynamic response of the RI underneath the fluorescence peaks. (Vertical scales are shown with arbitrary units on an 8-bit scale.) Horizontal blue bars on the graph indicate that the measurement was immediately preceded by 30 sec photo-activation, and their placement indicates the intersection of the analysis line with fluorescent foci.

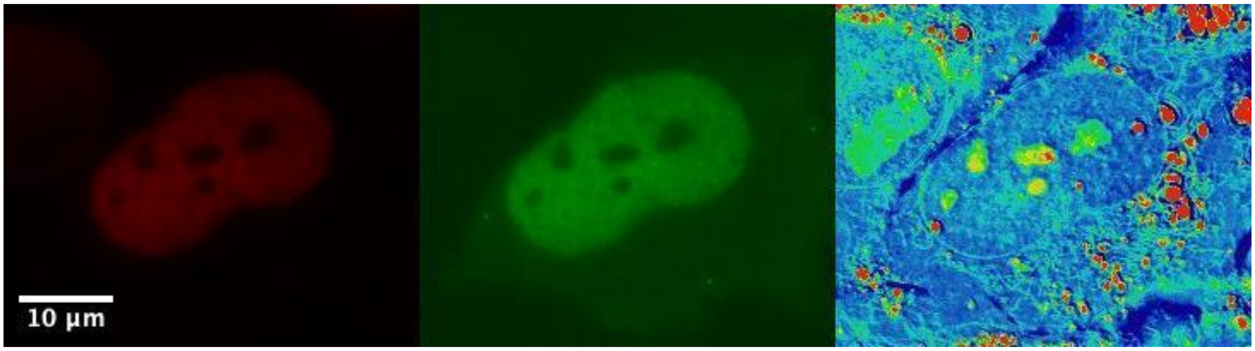

Movie S4: Image compilation of the Ft-mCherry, FUS-GFP, and the RI time series depicted in Figure 8. Images were recorded every 30 seconds. Saturated fluorescence frames indicate the periods of photo-activation, corresponding to the blue bars shown in the figure.
