# Supplementary material for: Refractive index as an indicator for dynamic protein condensation in cell nuclei
Source: Biophys Rep (N Y). 2025 Nov 1;5(4):100235. doi: 10.1016/j.bpr.2025.100235 (PMC12666831; doi:10.1016/j.bpr.2025.100235)
Supplement: Document S2. Article plus supporting material [file mmc6.pdf]

Biophysical Reports

ARTICLE

Refractive index as an indicator for dynamic protein condensation in cell nuclei

Orlando Marin,[1] Peter Kirchweger,[1] Arina Dalaloyan,[1] Yoav Barak,[2] and Michael Elbaum[1,*]
[1]Department of Chemical and Biological Physics, Rehovot, Israel and [2]Department of Chemical Research Support, Weizmann Institute of Science, Rehovot, Israel

ABSTRACT Protein condensation is the basis for formation of membrane-less organelles in the cell. Most famously, weak, polyvalent interactions, often including RNA, may lead to a liquid-liquid phase separation. This effect greatly enhances local concentrations and is thought to promote interactions that would remain rare in dilute solution. Synthetic systems provide a means to clarify the underlying biophysical mechanisms at play, both in vitro and in the cell via exogenous expression. In this regard, ferritin is a useful substrate, as its composition of 24 subunits with octahedral symmetry supports self-assembly by close packing in 3D. The conventional diagnostic tool for protein condensation is fluorescence imaging. In this work, we explore the use of refractive index mapping to detect states of condensation and decondensation. Using two related ferritin-based self-assembly systems, we find that refractive index is a sensitive indicator for reversible condensation. Surprisingly, refractive index indicates a rapid decondensation even when molecular dispersal kinetics are slow according to fluorescence. Conversely, in a photoactivated condensation where long activation results in slow decondensation kinetics, the refractive index provides reliable evidence for the physical state independent of fluorescence. The observations suggest a distinction between condensation to a sparse biomolecular network or to a material continuum that supports an optical polarizability distinct from that of the dilute phase in solution.

WHY IT MATTERS Holographic methods of refractive index mapping offer a label-free alternative to fluorescence imaging to study protein condensation. Here, we explore this possibility using two related ferritin-based constructs: a stable one-component system of supramolecular protein assemblies, and a two-component photo-inducible system involving interaction between intrinsically disordered protein domains (Corelets). In both cases, refractive index is sensitive to the state of condensation and reveals internal inhomogeneity that may be difficult to resolve by fluorescence. Moreover, the data suggest a distinction between a locally elevated molecular concentration as suggested by phase condensation and the condensed state as a material continuum reflected in an elevated dielectric polarizability at optical frequencies.

INTRODUCTION

Macromolecular condensation is recognized as an important mechanism in cellular biochemistry. Interaction between proteins and/or nucleic acids, whose average concentration is low in the cell, can be accelerated and regulated if brought into proximity by co-condensation into a common dense phase. This thermodynamic phase separation underlies the concept of membrane-less organelles, and the dense regions are commonly known as biomolecular condensates (1). Their state is often fluid (2), leading to the description as liquid-liquid phase separation. The fluid state facilitates molecular mixing and interaction within the condensate.

Mechanistically, phase condensation reflects the effect of weak but polyvalent interactions between binding partners. Many proteins are recognized for their marginal stability in solution, often leading to aggregation or organized self-assembly (3,4). This may involve a drastic structural rearrangement, for example, in amyloid formation (5) or in ordered self-assembly as occurs in cytoskeletal or flagellar filaments. Intrinsically disordered protein (IDP) domains are particularly prone to condensation due to their

Submitted April 24, 2025, and accepted for publication October 28, 2025.

*Correspondence: michael.elbaum@weizmann.ac.il

Editor: Jorg Enderlein.

https://doi.org/10.1016/j.bpr.2025.100235

© 2025 The Authors. Published by Elsevier Inc. on behalf of Biophysical Society.
This is an open access article under the CC BY license (http://creativecommons.org/licenses/by/4.0/).

weak and transient hydrophobic interactions, as well as to amyloid-like misfolding (6). Indeed, misfolding of common IDPs is a hallmark of many neurodegenerative diseases (7). RNA binding is a common motif for promoting heterogeneous interaction and condensation; the disordered nucleic acid provides a flexible substrate for protein interaction. On the other hand, phase separation may be viewed more strictly as a self-assembly phenomenon. For example, extended supramolecular structures can be constructed from designed peptides of chosen shapes and forms (8).

In a similar spirit, self-assembly of a hybrid ferritin fusion to self-dimerizing fluorescent proteins (FPs) led to the formation of spherical bodies (9). These supramolecular protein assemblies (SMPAs) form spontaneously in living tissue culture cells upon expression of the constituents from a transfected plasmid. Assembly could be directed to the cell nucleus by inclusion of a nuclear localization signal (NLS) sequence to the FP. The symmetry of the ferritin core with 24 subunits provides a convenient building block for self-assembly because a sphere in a close-packed pile has 12 nearest neighbors; therefore, weak interactions at the molecular level can induce self-assembly at a much larger scale. When formed in the cell nucleus, evidence of a crystalline structure was observed. The overall structure was often hollow or alveolar, suggesting a sintering of smaller subassemblies, but not a liquid-like state. Formed in bacteria, on the other hand, the assemblies showed no long-range molecular order (10). Self-assembly depends on antiparallel dimerization at a hydrophobic patch of amino acids common to green fluorescent protein and derivatives (Ala206, Leu221, Phe223), and it can be suppressed by the mutation A206K. The hydrophobic patch could instead be replaced by a cysteine-alanine combination, in which case the self-assembly in nuclei was triggered by addition of a thiol oxidant to the cell culture medium (11). Such oxidation-induced assemblies were filled rather than hollow and could be seen to sinter, suggesting a liquid-like state.

A connection between protein self-assembly and condensation was drawn by creating a light-induced linker between the ferritin core and an IDP from the fused in sarcoma (FUS) protein in what was named the "Corelet" system (12). Ferritin was fused to an iLID (improved light-induced dimer) domain via an FP in one color, whereas a condensing intrinsically disordered region (IDR) domain was fused via an FP in a second color to SspB. iLID and SspB dimerize under blue light illumination, leading to self-assembly of a network of ferritin proteins linked by interactions between the concentrated IDR. This model system elegantly recapitulates many of the salient features of biomolecular phase separation. Using FPs of distinct colors and careful fluorescence intensity calibrations, the phase space for condensation could be mapped semi-quantitatively (12).

In this work, we explore the use of optical refractive index (RI) as a diagnostic tool for condensation. Quantitative phase imaging has been used for measurements in vitro without the concerns and constraints associated with fluorescent labels (13). The power of RI mapping of cells has also been demonstrated (14–18) by a number of phase-sensitive and holographic microscopy techniques, summarized in a recent review (19). Here, we employed the commercial 3D CellExplorer system (NanoLive, Switzerland). All measurements were made on live cells, as chemical fixation, whose nature is amino acid cross-linking, risks altering the delicate balance of entropy and interaction that leads to phase separation in the first place (20). We found that the condensed phases were more refractive than the dispersed. Moreover, the one-component SMPA displayed a higher RI than the two-component Corelets, whose cross-links are more sparse. Quite unexpectedly, we found that the fluorescence intensity could remain high in defined intranuclear spaces even when the RI indicated loss of condensation. Also unexpected were observations of persistent condensation after sufficiently long blue light exposure, even after the blue light was extinguished, and of a further compartmentalization within the dense phase of the Corelets under ferritin-rich conditions. These results suggest that the condensation behavior is richer than the simple biphasic coexistence that is often presumed as a starting point for analysis and also point to a difference between the phase separation reported by fluorescence intensity and the dielectric response of a condensed phase as detected by RI.

MATERIALS AND METHODS

Plasmid preparation

The one-component SMPA plasmid for NLS-citrine-ferritin in the pcDNA3 vector was described in a previous publication (9) and used as is after amplification. The two-component Corelet plasmids for NLS-iLID::mCherry::FTH1 and FUS_N::EGFP::SspB (12) were recloned into pcDNA3 from lentiviral vectors received from the Brangwynne lab. Sequence maps are provided in the supporting material. Plasmids were amplified in DH5α cells and purified using the Promega PureYield kit. Plasmid maps are shown in Fig. S1 and are available upon request.

Cell culture and transfection

U2-OS and HFF-1 cells were obtained from ATCC. Cell cultures were maintained under 5% CO_2 at 37°C in DMEM medium (Dulbecco) with 5% fetal calf serum (Biological Industries, Israel).

DNA transfection was performed on cultures at approximately 75%–90% confluence using JetOptimus (Polyplus), following the manufacturer's protocol. SMPAs in Fig. 2 were induced by transfection with 1.3 μg DNA. Dual transfections at various ratios were performed in order to induce Corelet formation exploring the variety of structures and sizes. DNA stoichiometry Ft:FUS included a ratio of roughly 1:3 (Figs. 3, 4, and 5), a high Ft:FUS plasmid ratio to generate larger condensates (5:1 in Figs. 6 and 7, respectively), and a small ratio to produce small condensates (1:8, Fig. 8). In the transient transfection protocol, the plasmid concentrations are only one factor governing protein expression levels; they set a trend, but cells had to be identified for study individually.

Refractive index measurement

RI mapping was performed in the 3D Cell Explorer-fluo microscope (NanoLive, Switzerland). The microscope is equipped with an on-stage incubator to maintain an environment of 5% CO_2 at 37°C, as well as fluorescence illumination for green and red emissions. The microscope is controlled by the manufacturer's "Steve" software, which also provides tools for preliminary visualization. The instrument setup includes a preset self-calibration routine. We found that the reported values were lower than expected and somewhat variable, so we established a second stage of calibration based on RI of polystyrene (Polysciences, 3.0 μm, $n = 1.600$) and silica (Bangs Laboratories, 1.1 μm, $n = 1.440$) nanobeads, and water ($n = 1.333$). The resulting linear regression was applied after export of the raw images in floating point format from Steve to Fiji (21). This protocol yielded a RI close to 1.45 for the intracellular lipid droplets, as expected for triglycerides. Further image analysis was performed in Fiji after export of image data from Steve in floating point format, and quantitative analysis was performed using Origin (OriginLab, USA).

The homographic RI analysis produces volume maps of typically 96 slices, with voxel dimensions 183 × 183 × 482 nm. Interspersed with the RI map, 2D fluorescence images were recorded by the wide-field camera with the same pixel size and exported from "Steve" as single 8-bit tiff images. The microscope records such images for one or two colors at a single focus. No postprocessing of the FM images was applied other than intensity adjustments. Due to the limited 8-bit dynamic range and the need to set the illumination and exposure parameters according to the relatively dim de-condensed fluorescence, the FM channels often became saturated after condensation. For visualization, the fluorescence images were exported as independent tiff files and, where appropriate, overlayed onto the best matching slice of the RI volume map. Finally, an RGB composite image was created with the desired lookup table.

Photoactivation

Photoactivation of the Corelets was performed using a stand-alone LED illuminator with a 22-mm aperture, 470-nm wavelength, LED Type A (3W) with 200-mW-rated output, bare-wire connector (LCS-0470-03-22, Mightex, CA, USA), controlled by an SLA-series two-channel LED Driver (SLA-1000-2, Mightex). The illuminator was mounted diagonally approximately 12 cm from the specimen dish. The LED was focused by a lens to cover an area of 2.6 cm diameter on the dish. The power density can therefore be estimated at 0.04 W/cm^2, which is orders of magnitude lower than typically used for confocal microscopy. A brief 10-s exposure was used to identify cells susceptible to condensation.

Statistics

The RI values from the calibrated images were extracted and exported to an Excel file, from which violin plots were created using the Python seaborn library defaults. In Figs. 2, 5, and 6, the violin plot includes a small boxplot, in which the white line represents the 50th percentile (median). The bottom and top edge of the box represent the 25th and 75th percentiles. Violin plots with the actual data points overlaid are available in the Supporting Data. In Fig. 3, we plotted the actual data points. From the RI maps displayed in Fig. 8, we extracted the median and maxima of each region of interest (ROI), plotted these values (rounded to 0.005) as purple dots, applied thresholding as shown in Fig. 8 *B* (median) and Fig. S5 (max), and summarized the number of ROIs in Table S1.

RESULTS

The protein systems under study are displayed in Fig. 1. We will refer to the constitutive single-component system as SMPA (Fig. 1 *A*) and the photo-activated, two-component system as Corelets (Fig. 1 *B*). Both form in the cell nucleus. SMPAs begin to appear approximately 4 h after transfection, whereas Corelet components require a somewhat longer expression in order to form condensates upon blue light illumination. Both were examined typically 12–24 hours after transfection. The primary tool in the study is a holo-tomographic microscope (3D Cell Explorer-fluo, NanoLive, Switzerland), which creates a volume map of the RI using highly tilted illumination recorded in multiple projections around an axis. We found that the RI measurements show high precision and remarkably low noise in a homogeneous medium. Numerical accuracy was unsatisfactory, however, and quantification required several calibration steps, as described in the materials and methods. The microscope is also equipped for wide-field fluorescence illumination and with an environmentally controlled chamber for extended live-cell observation.

An RI map of the SMPA appears in Fig. 2, with two adjacent cells in Fig. 2 *A*. The nuclear envelopes are visible in the images. SMPAs are identified unambiguously by fluorescence, and contrast in the RI image is very strong. Several clusters of adjacent spheres are seen. Since their growth occurs over many hours, at the rate of protein expression, they appear to sinter as particles but not to fuse as a viscous fluid. The inset also hints to some internal structure that is poorly resolved. This is consistent with the alveolar structure reported in the original work by confocal fluorescence and transmission electron microscopy (9). The distribution of RI values could be quantified by using the fluorescence image as a mask overlaid on the RI image. An extreme example of internal structure appears in Fig. 2 *B*, where the SMPAs appear in the RI image as thin spherical shells. The fluorescence signal was easily saturated, on the other

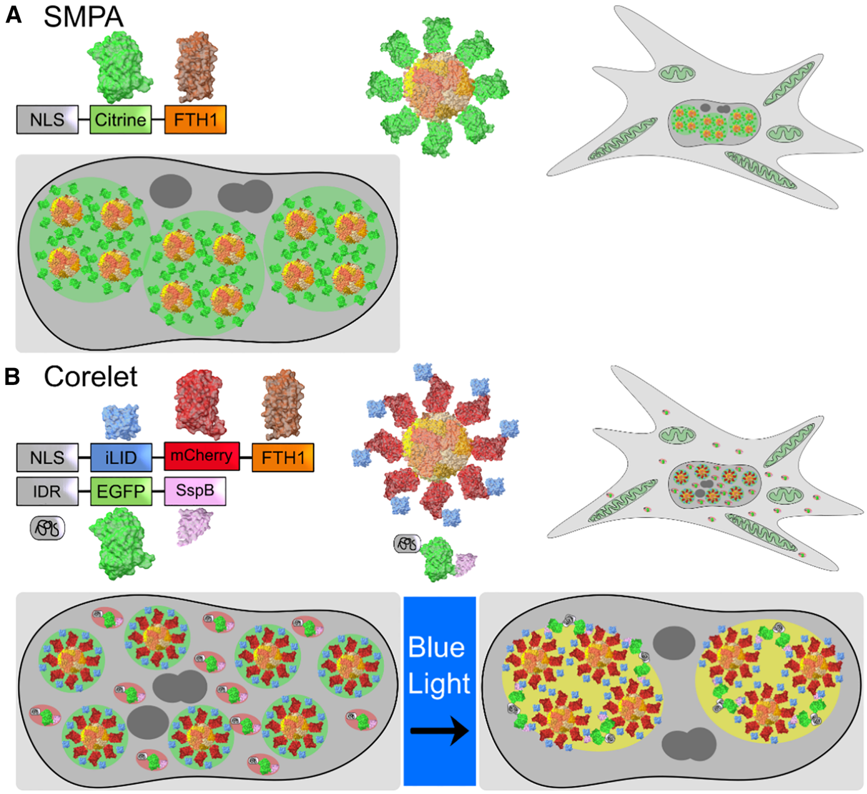

FIGURE 1 Overview of the supramolecular protein assembly (SMPA) and Corelet systems. (*A*) Schematic representation of the SMPA. Spontaneous self-assembly is driven by antiparallel dimerization of the fluorescent proteins. The 24-mer octagonal structure of ferritin supports formation of close-packed aggregates. (*B*) Schematic overview of the Corelet system. Dimerization is driven instead by light-induced linkage via iLID/SspB of ferritin cores to an intrinsically disordered region (IDR) of FUS, which is itself prone to liquid-liquid phase separation.

hand, due to limited dynamic range of the 8-bit recording, and the central hole does not appear. Saturated fluorescence is a common problem in the field, as a large dynamic range is required in order to display both the bright condensate and the dim dilute phase. Moreover, out-of-focus fluorescence will make the condensates appear larger than they truly are. A line profile across the hollow sphere shows the high RI concentrated at the SMPA rim. Examples in other cell types appear in Fig. S3. Given the dimensions of a single voxel, it is likely that the RI measurement is somewhat underestimated due to cross-coverage with the surrounding nucleoplasm. Nucleoli are also seen in the RI but not in the fluorescence. Notably, the RI of the nucleoli is lower than that of the SMPA.

Preliminary observations of Corelets showed a similar correlation of fluorescence with RI, but analysis was more challenging due to the lower RI contrast and smaller size of the condensates. Fig. 3 establishes the visualization and analysis strategies. It displays the fluorescence in the two individual colors, red and green, for ferritin (Ft) and FUS, respectively, as well as a composite image in yellow. A threshold imposed on the fluorescence intensity delineated the regions of high protein concentration as ROIs in Fiji, shown outlined in purple. The ROI boundaries were eroded morphologically (automatically in Fiji) in order to compensate for the spread in the fluorescence signal that originates from planes out of focus. (Erosion by zero, one, or two pixels was done on a case-by-case basis.) These ROI were then used as selection masks for the corresponding image locations in the RI map. Note that the RI map is volumetric and resolved in depth. A single 2D slice is used for display with an inverted lookup table (higher index dark), as well as a blue to red ("physics") color scale. For quantification, several slices were chosen so as to limit to regions within the nucleus, and a maximum intensity projection was applied in order to match the volume from which the fluorescence emerges. (The method is prone to error when more refractive elements, such as nucleoli, encroach into the contour-defined area in planes above or below the condensed body of interest. In such cases, the measurements must be discarded.) Corelet condensates are always more refractive than the nucleoplasm (Np) and chromatin background, but unlike the SMPA, they are typically less refractive than the nucleoli (Nc). The measured RIs display a significant spread but appear to be independent of size, which ranged from 4 to 40 pixels (0.033 μm^2/pixel).

With the quantification protocol in hand, we first recapitulate the dynamic behavior of the Corelet system. Fig. 4 (also Videos S1 and S2) shows a cycle of condensation, dispersal, and recondensation upon transient blue light illumination. A rapid illumination

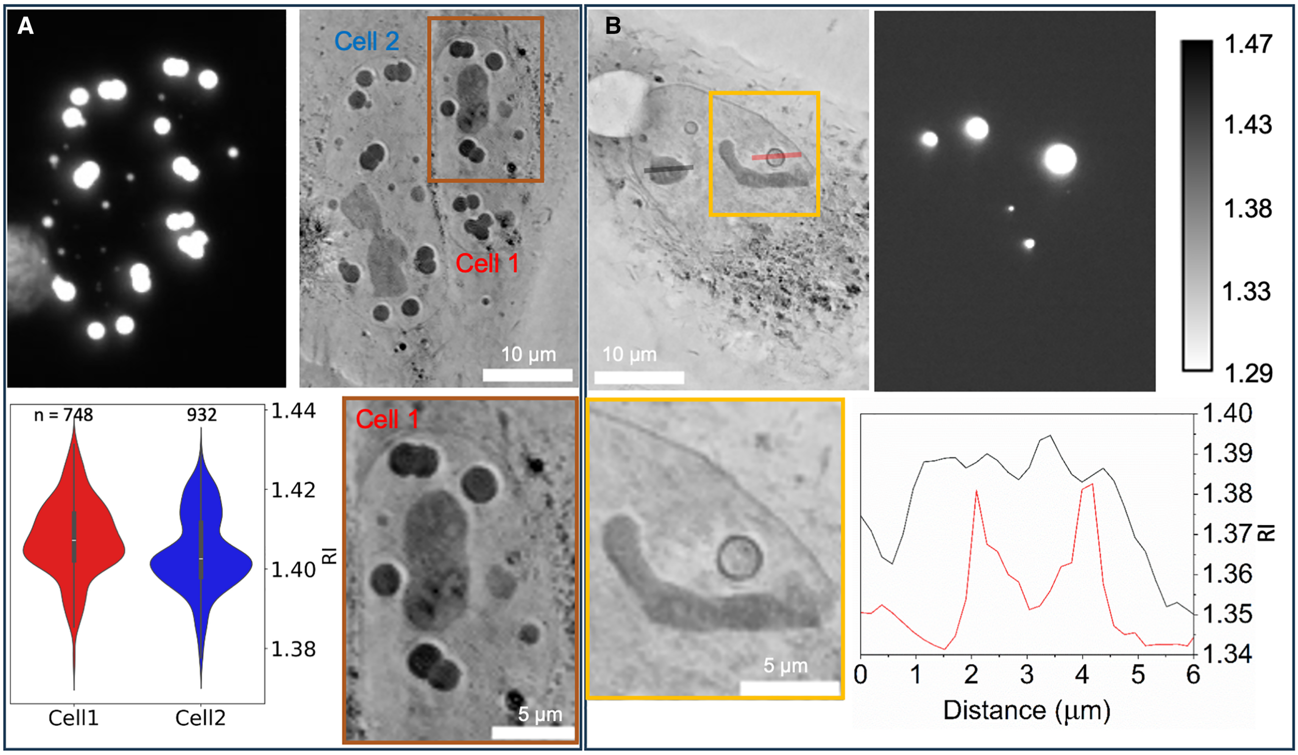

FIGURE 2 Fluorescence and refractive index measurements of one-component ferritin SMPA. (*A*) Both large and small condensates are seen in the fluorescence, and the corresponding locations show a high RI. Note the clusters of abutting SMPA; they appear to sinter but not fuse, suggesting a solid rather than liquid physical state. Note too, the variety of contrast levels represented, as indicated in the violin plots. The small boxplots show the 25th, 50th, and 75th percentiles: lower edge, white line, and upper edge of the box, respectively. The same violin plot with all data points is shown in Fig. S2. (*B*) SMPAs may also take a hollow shell form, seen in the RI even when unresolved in the fluorescence image. Additional examples of SMPAs in the cell nucleus of U2OS and HFF cells appear in Fig. S3.

(10 s) was used first to find the transfected cells in which condensation occurs. Subsequent cycles with 30-s illumination showed a rapid response of the RI signal, whereas the fluorescence showed a hysteresis with the signal intensity, remaining elevated near the former condensate locations. An example of the discrepancy is highlighted by the oval shape, shown in purple; compare points T2 to T1, or T6 or T7 to T4.) After repeated cycles, the persistent fluorescence became more clearly defined; compare T10 to T4. (The white circle highlights a nonfluorescent nucleolus.) A line scan across two Corelets and a nucleolus highlights this behavior in Video S3. Although the fluorescence and RI are high directly after blue light illumination (e.g., T1, T3, T4, T8), in the following timepoints (e.g., T5–T7, T10) the fluorescence peak remains high, whereas the RI peak drops. Fluorescence intensity reveals the local protein concentration. The condensation likely created or exaggerated 3D voids in the chromatin (22), away from which diffusion would be slow. RI, on the other hand, relates to dielectric polarizability at optical frequencies. It reflects the bulk material continuum rather than the concentration of isolated molecules. Thus, the continuum, condensed state is rapidly lost even though the local protein concentration remains high.

Before blue light exposure, the fluorescence of both Ft and FUS was often heterogeneous, again consistent with a heterogeneous distribution of chromatin in the background (23). Punctate fluorescence concentrated in the brighter diffuse areas. Unexpectedly, after an extended illumination of 20 min with blue light, the condensates did not disperse over a period of more than 30 min (Fig. 5). Fluorescence remained punctate and intense, and the RI also remained uniformly high at the same locations. This indicates an annealing process and suggests that the internal structure of the rapidly responding condensates differs from that of the annealed ones. A possibility to consider is the transition to a gel state. However, the FUS moiety is interspersed with the ferritin. Given the two-component assembly mechanism, one may expect that the internal FUS concentration may reconfigure in a manner that imparts stability to the composite assembly.

In order to test the stability, we increased the amount of expression plasmid in an effort to create larger condensates and illuminated for a shorter

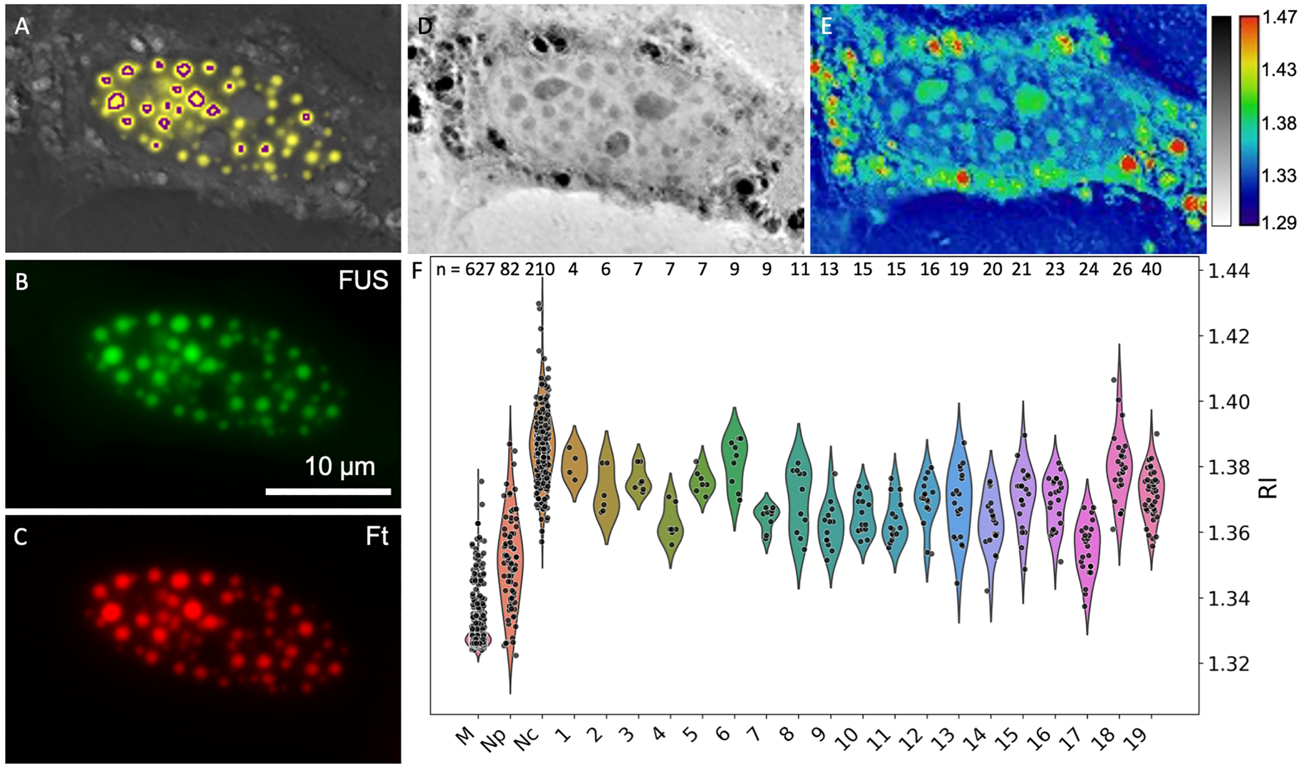

FIGURE 3 Quantification of RI for two-component Corelet condensates. (*A*) FUS and ferritin composite image appears in yellow, overlayed on the RI image. The areas of condensation are delineated according to an intensity threshold, eroded morphologically to compensate for fluorescence haze effects, and outlined in purple. (*B* and *C*) The two fluorescence channels appear in green (FUS, *B*) and red (Ft, *C*), respectively. (*D* and *E*) The RI images are shown in grayscale (*D*) and false color (*E*) for enhanced visibility. Highly refractive features in the cytoplasm are lipid droplets. (*F*) The plot shows a distribution of RI values extracted from the surrounding medium (M), the nucleoplasm (Np), the nucleoli (Nc), and the 19 regions of interest (ROIs) that delineate the Corelet condensates. The numbers on top of the violins indicate the number of pixels per component/condensate. Condensates smaller than four pixels were removed from the analysis.

extended period of 10 min. Results of this assay appear in Fig. 6. At the 5-min time point the condensates remain, but after 6 min in darkness, they began to disperse according to the RI contrast. Quantification showed a skew of the RI distribution to lower values, as seen in the histogram. The fluorescence still indicated a condensed state, albeit with a subtly different distribution. A 20-s reillumination restored the condensed state at the 7-min time point, as seen both in the image and in the histogram of RI values. At 8 min, the image reveals the sintering of nearby spheres into elongated shapes; the histogram shows a rearrangement of values at the peak but a remarkable overlap at the upper tail. The flow observed suggests that the dark-persistent condensates do remain in the state of a viscous liquid.

Under the same transfection conditions, another cell displayed a spinodal decomposition rather than condensation to spherical droplets. This is consistent with a high level of protein expression, as described in the original Corelet publication (12). A striking and hitherto unconsidered feature was observed, as displayed in Fig. 7. Small, much denser spheres or shells appear within the condensed phase. The RI of these dense bodies is very similar to that of the one-component SMPA, suggesting an interpretation as a ferritin-dense core and ferritin-poor shell surrounding it. Moreover, the hollow shell or alveolar shape suggests that these core bodies do not flow as a liquid.

At the opposite extreme in concentration, an example of very small condensates appears in Fig. 8. This presented a challenge for the analytical workflow. Due to the optical effects (diffraction limit and defocus), the size represented in the fluorescence image is necessarily larger than the true size in the sample. Therefore, the mask defined by the fluorescence overestimates the relevant area severely. In order to detect such condensation, we present all the RI voxel values within the fluorescence-defined mask (without erosion) and focus attention only on those few pixels taking values measurably above the background. These high-valued pixels, indeed, responded to blue light illumination as expected for repeated short exposures. In the RI images, these outliers can be seen most effectively on the color scale in the insets displayed. (The highly refractive nucleolus, dark

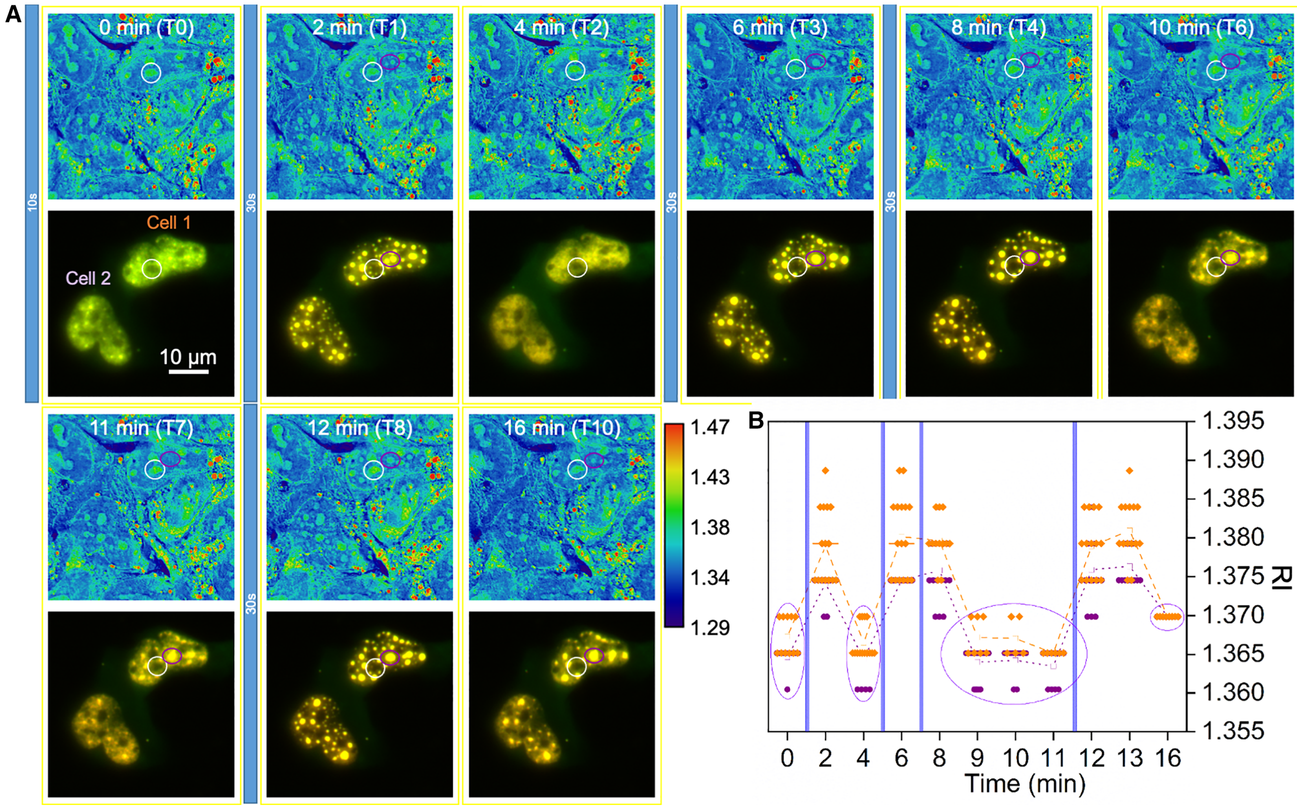

FIGURE 4 Condensation tracks photoactivation. (*A*) A time series over 16 min (T0–10) shows that refractive index (*false color*) and fluorescence (*composite in yellow of the FUS and Ft*) track the condensation induced by blue light illumination. Illumination periods are indicated by vertical blue bars. With repeated cycles, the fluorescence does not diffuse completely, but the RI indicates complete condensation and decondensation. For specific comparison a nucleolus (*white circle*) and a Corelet (*purple oval*) are highlighted. Videos S1 and S2 show the full field of view and the FUS-GFP, Ft-mCherry, and RI ("physics" LUT) channels side by side. Video S3 shows RI ("physics" LUT), the FM composite on the RI in greyscale, and a line plot as time series, respectively. (*B*) A plot of RI measurements according to the method of Fig. 3. ROIs are defined anew with each condensation and used for the remainder of the cycle to determine the RI. Values have been rounded off at steps of 0.005 for clarity.

in fluorescence, serves as a spatial anchor.) Compare, for example, the 8.5- and 10-min time points, where the Corelet condensates appear almost as noise in the earlier measurement but can be identified clearly by correlation with the fluorescence. The peak values fall in the range described in Fig. 4, close to 1.380, so the internal structure of the very small condensates is likely similar to that of the larger ones.

DISCUSSION

Protein condensation in cells, and particularly the phenomenon of liquid-liquid phase condensation, has attracted enormous attention as a mechanism for biochemical regulation in vivo. The biological examples are broad and varied. Synthetic systems offer a more controlled platform for investigating basic biophysical principles. In this light, the two ferritin-based systems offer a bridge between the concepts of kinetic self-assembly and the thermodynamics of phase condensation. Although simple theoretical considerations posit coexistence between individually sparse and dense phases, the observations here indicate an evolution of the dense phase over time. Specifically, illumination for 20 min or longer resulted in persistent condensation lasting more than 30 min in the dark. Illumination for 10 min resulted in condensation lasting approximately 5 min, with slow evolution of the shapes that suggests retention of the viscous liquid-like physical state. Illumination for 30–60 s confirmed the reversibility reported previously. We may, therefore, ask what internal changes occur in converting the transient to the persistent state.

In the one-component SMPA, each ferritin subunit is hybridized to a dimerizing unit in the FP. Thus, each ferritin protein holds 24 potential linkers. The resulting SMPA is stable and solid phase, as judged by the presence of internal voids. The high density is reflected in the high RI values, equal to or typically greater than those of the nucleoli. The Corelet condensates show a lower RI than the SMPA, typically lower even than that of the nucleoli. RI values also

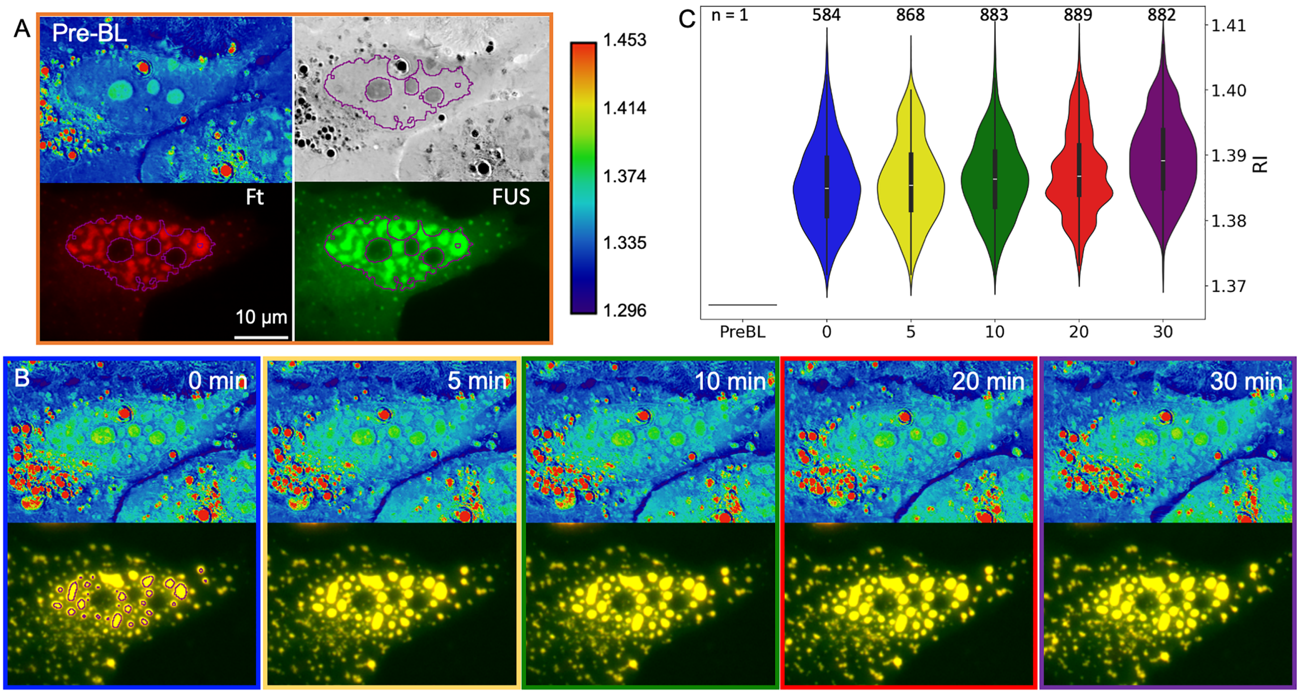

FIGURE 5 Long activation time induces persistent condensation. (*A*) Before blue light exposure (pre-BL) the cell shows no indication of condensation by RI. Fluorescence is distributed unevenly within the nucleus, however, consistent with exclusion from regions occupied by dense chromatin. (*B*) A time trace of images recorded after 20-min continuous activation, showing that the condensates do not disperse. The RI ROI masks are generated for every time point individually and shown as an example in the 0-min image (*purple lines*). (*C*) Quantification of the RI in condensed regions shows that the RI remains constant for at least 30 min. Violin plots are described in Fig. 2. (Plot showing all the data points appears in Fig. S2). See Fig. S4 for an additional example of long activation resulting in persistent condensation.

vary more significantly from one condensate to another, even within the same cell. This may reflect a heterogeneity in the local FT:FUS stoichiometry, which will depend on transfection DNA concentration, transfection efficiency, and levels of protein translation. Only the first is under direct control, and the results shown here appear to explore the limits of the phase coexistence boundaries (12).

The dark-persistent condensation upon long activation was unexpected. The RI of long-illuminated, persistent Corelet condensates was not significantly higher than that of the transient ones. Persistent condensation was described almost anecdotally in the original Corelet reference (12), where it was noted that high blue laser intensity in the context of photobleaching might damage the iLID domain of the photoactivatable linker. In the present observations, the persistence resulted from a long activation duration, but the same intensity was far lower than that employed in confocal microscopy and was not sufficient to cause noticeable photobleaching in any of the experiments. Another suggestion might be to invoke the tendency of the FUS IDR domain to transform to a gel state upon aging (24). Irreversible aggregation was indeed observed for the same FUS protein domain in a one-component photoactivatable predecessor to the Corelet system (25); these aggregates retained their irregular shapes upon sintering, counter to expectations for a liquid phase. In the present case, the FUS moieties would be interspersed between ferritin cores. A more stable, gel-like interaction between the neighboring FUS might nonetheless form during the long photoactivation, leading to a solid cast of FUS surrounding the ferritin. In the subsequent period of darkness, the equilibrium affinity of the iLID-SspB interaction should drop drastically, but the contact may not actually rupture if the two components are held together externally. For the 20-min photoactivation, the condensates were stable for at least 30 min, whereas for the 10-min photoactivation, a partial decondensation was detected after 7 min. Recondensation after a short blue light exposure confirms that the dark-persistence is not due to inactivation of the iLID. It is also unlikely that the FUS domain reached an irreversible prion state on this timescale, especially given the maintenance of a spherical shape and the absence of any detectable change in RI.

We consider next the use of RI as a diagnostic for protein condensation. The measurement is compatible with live imaging and provides very useful depth

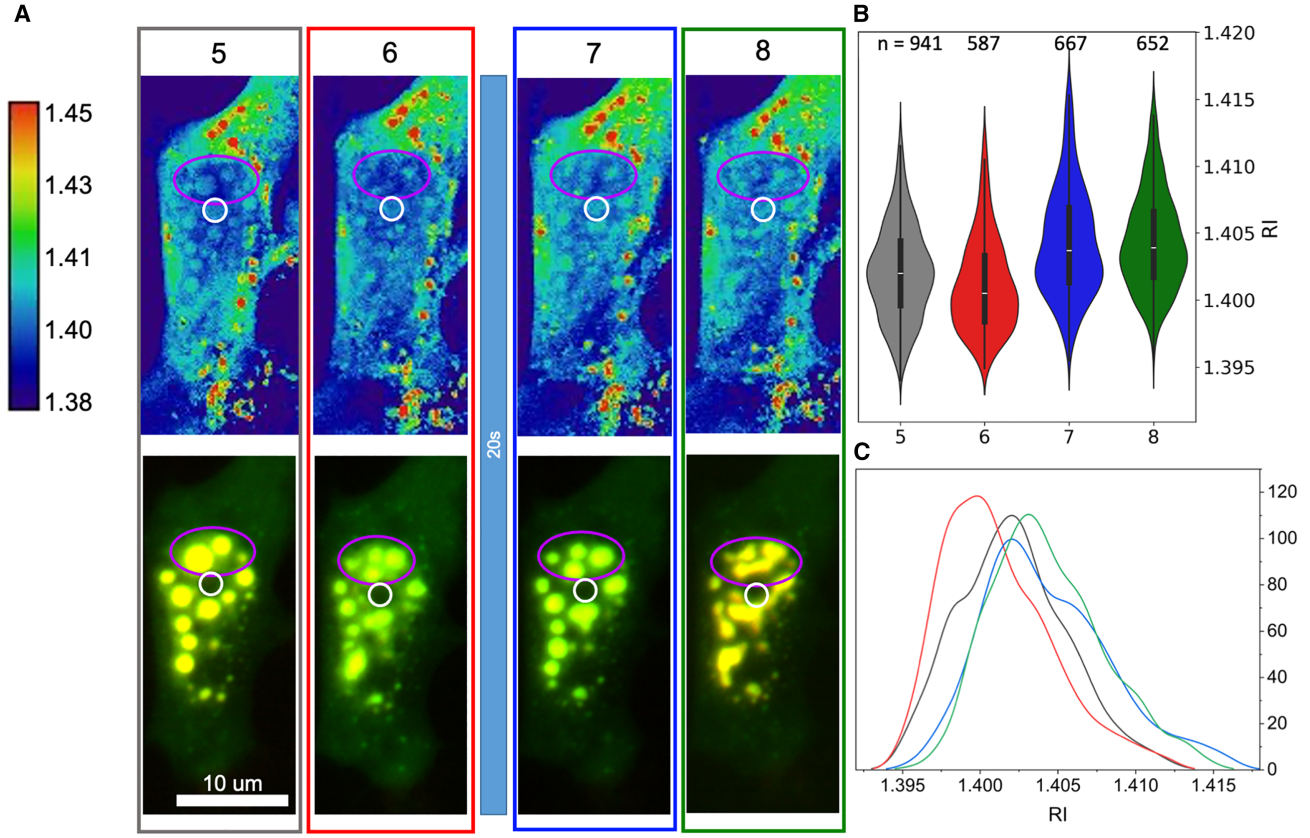

FIGURE 6 RI indicates marginal condensation persistence. The cells were first illuminated continuously for 10 min (not shown) and then kept in darkness (other than rapid illumination for fluorescence imaging with exposure of 1 s at 30-s intervals) for 6 min. (*A*) Decondensation is clear by 6 min in the RI but not in the fluorescence. A subsequent illumination for 20 s restores the condensation. By 8 min, the condensates are seen to fuse, suggesting the fluid state. Purple and white outlines highlight an example of the decondensation imaged in RI and the nucleoli, respectively. Quantification of the RI measurements using (*B*) a violin plot and (*C*) histograms show subtle changes during this episode, with a reduction in RI between 5 and 6 min and then restoration at 7 min. This is seen in the point distribution as well as the histogram of RI values, with corresponding colors indicating the time points. The violin plots are described in Fig. 2. The violin plot with data points overlaid appears in Fig. S2.

resolution without the complications of fluorescence or confocal imaging, as well as lower risk of phototoxicity. Also, although fluorescence may be required for molecular identification, there is always a risk of interaction induced by the FPs themselves; indeed, FP dimerization is the driving force for the SMPA growth. In this work, we took a correlative approach, interleaving fluorescence and RI measurements on the same specimens in real time. Unfortunately, the two capabilities were not well balanced in the instrument at hand, with the fluorescence imaging being rather primitive in comparison to the very sophisticated 3D reconstruction of the RI map. Nonetheless, we saw that the RI responds specifically to the condensed state, whereas the fluorescence measures, in practice imperfectly, only the local protein concentration. We can recommend that quantitative fluorescence should be a future design goal for correlative imaging. An elegant recent publication combined confocal fluorescence with a custom-built instrument for quantitative phase imaging to address conservation of the protein concentration ratio between nucleus and cytoplasm (26).

Perhaps the most surprising observation was the apparent discrepancy in dispersal kinetics upon removal of the condensation-inducing photoactivation. Repeated cycles of Corelet condensation and decondensation in the nucleus appear to create a space from which diffusion is slow. As seen in Fig. 4, and especially in Video S2, the RI rises and falls back to background level immediately when the photoactivation is applied and removed, yet the fluorescence remains locally elevated. In addition, recondensation occurs in the same locations repeatedly. Given the instrumental limitations to two fluorescent channels, it was not possible in the present work to visualize the chromatin simultaneously with the Corelets here. Formation of voids in chromatin due to protein condensation has been reported previously, however (22). Furthermore, condensation dynamics and phase

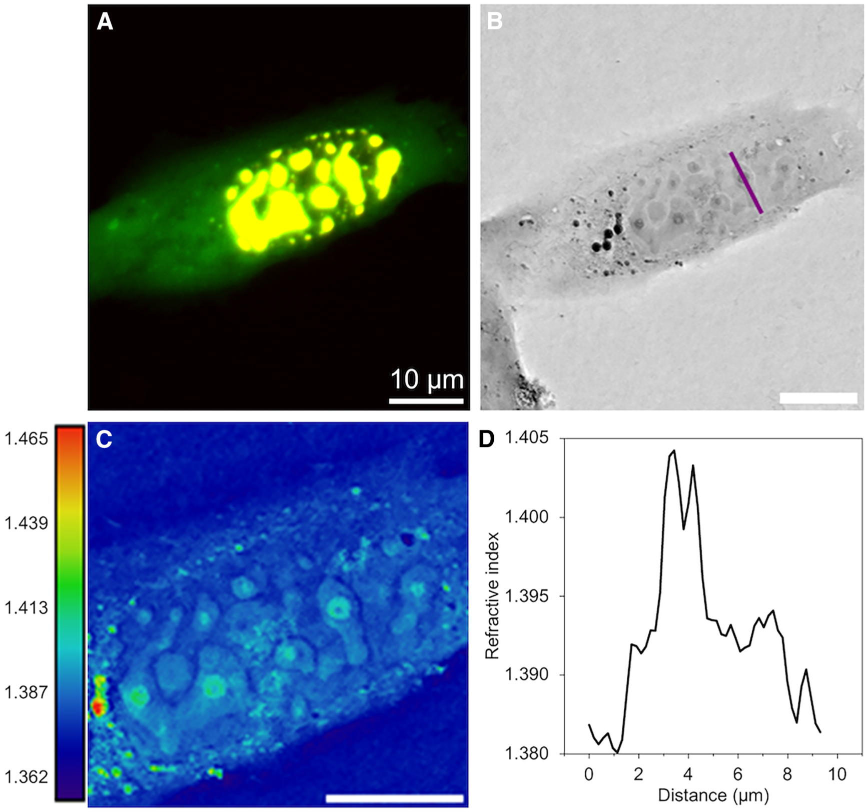

FIGURE 7 Very large condensates show an internal compartmentalization. (*A*) Fluorescence shows a typical pattern of condensation with high level of protein expression, with nonspherical shapes and apparent fusion. (*B* and *C*) The RI images show identical shapes but with much better resolution. The shapes are especially visible in the color map presentation. A very dense sphere appears within several of the condensates, often with an internal hollow core as highlighted by a line plot (*D*). The RI of the surrounding condensate is comparable to or slightly less than that of the nucleoli, but the RIs of the cores are much higher and recall those of the SMPAs seen in Fig. 2. Scale bars, 10 µm in all images.

behavior are sensitive to chromatin heterogeneity (23). A similar inverse relation between chromatin density and protein condensation was observed using an oxidation-inducible variant of the one-component SMPA (11). Also notable is the ability of condensates tethered to chromatin elements to apply mechanical forces (27).

Upon longer activation, as seen for example in Figs. 5 and S4, the condensates are stable, and both fluorescence and RI signals appear constant over time. With an activation of intermediate duration, seen in Fig. 6, the RI proves more responsive and reliable as a diagnostic of Corelet condensation. The RI can also detect structure within the condensate, for example the dense cores in Fig. 7 or the hollow centers for the SMPA in Fig. 2, where there is also a hint of inhomogeneity within the densely filled bodies. Such internal structures would appear to be inconsistent with a liquid state of matter. It should be very interesting to explore the ferritin-based condensates using a remote micro-rheological technique such as Brillouin microscopy (28). For very small condensates, as seen in Fig. 8 (also Video S4), the RI puncta covering only a single or very few pixels are very difficult to identify from noise without the fluorescence signal, yet the RI is a more reliable indicator of their state of condensation.

The relation of protein concentration in solution and RI has been analyzed in the context of a mixing model based on independent contributions of the constituent amino acids (29,30). This relation is described as a RI increment, or derivative dn/dc, and the mass density or concentration in solution is extracted from the linear proportionality. The model has been extended to consider hydration shells and protein structure (31), as well as more complex molecular composition in solution (28,32). Here, we observe a dramatic effect on RI of the state of condensation (i.e., the transformation of isolated macromolecules into a material continuum) without a similarly dramatic change in concentration of the condensing proteins. The RI is a measure of dielectric polarizability at the optical frequency. Modeling of such polarization as an additive contribution of isolated dipoles is akin to a noble gas approximation, which ignores the possibility of extended electronic excitation and correlation at longer length scales. Thus, the molecular connectivity and linkage in self-assembly is likely to play a crucial role, because the RI returns to precondensation values while the local concentration remains high, according to fluorescence. It is quite possible that the dispersing FPs displace the nonfluorescent background such that the total average concentration remains similar, before and after the

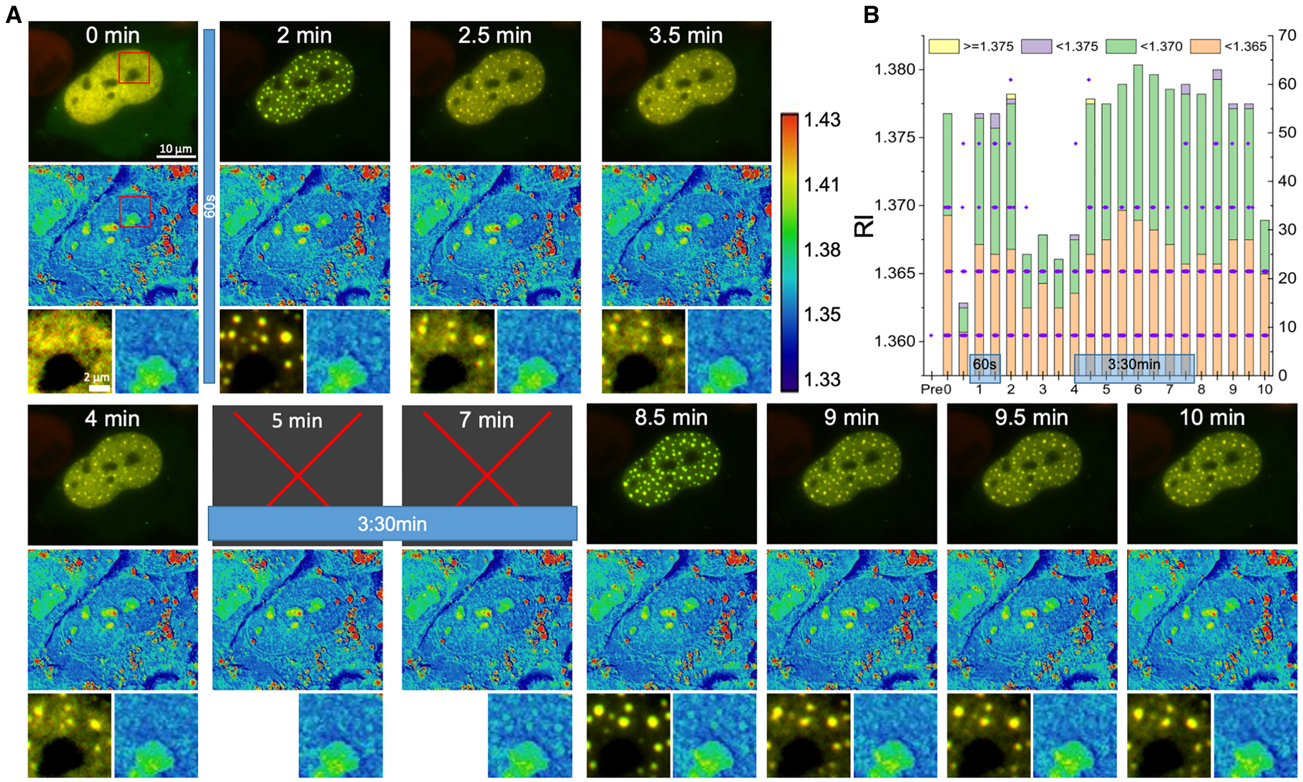

FIGURE 8 Very small condensates have refractive indices similar to larger ones. (*A*) Condensation induced by 60-s illumination is seen clearly in the fluorescence. Corresponding points appear in the RI images, which peak and then dissipate, but they cover only one or a few pixels. Essentially the same behavior is seen for 60-s and 210-s illuminations. Zoom-ins are taken from the area highlighted with the red box in the first panel (0 min). Scale bars represent 10 μm in the FM images and 2 μm in the zoom-ins. Video S4 shows the entire time series at 30-s intervals for the Ft-mCherry, FUS-GFP, and RI ("physics" LUT) channels, respectively; the flashing points in the RI color map are more noticeable there. (*B*) The quantification method of Fig. 3 was modified so the median was plotted for each ROI (*purple points*, values rounded for clarity), together with bars showing the number of pixels below threshold values (*orange for below 1.365, green for 1.365 to 1.37, purple for 1.37 to 1.375, and yellow for greater 1.375*). Clearly the RI values increase during periods of illumination and decrease after, but this is difficult to follow in the fluorescence alone. The number of median values in each category in (*B*) (i.e., the number of evaluated condensate locations) is shown in Table S1, and the same representation for the max values of each ROI is shown in Fig. S5.

condensation event. The elevated RI, on the other hand, is sensitive to the molecular connectivity of the Corelet components as well as their local concentration. This indicates that extrapolation of mass density from RI measurements of condensates, rather than solution, should be approached with some caution.

Another recent work reported on RI measurements of phase separation of nucleoli, as well as heterochromatin, nuclear speckles, and cytoplasmic stress granules (15). Our observations of nucleoli as dense condensates are very similar, and we too saw no hint of nuclear speckles or other nuclear protein condensates. (We did not induce or examine for cytoplasmic stress granules.) Quantitatively, our numerical measurements for the nucleoli are somewhat higher. This may reflect a difference in calibration protocol or the measurement technology, as our numbers for lipid droplets are also higher than a previous report (14). We consider this a minor discrepancy, however, since we do not aim to quantify mass density, and we use the nucleoli and lipid droplets as an internal standard against which to compare the synthetic protein condensates. Notably, the one-component ferritin SMPA, in which the hybridized linkages may saturate in close packing, showed a much higher RI than the Corelet condensates, wherein the linkers are more sparse. The RIs of SMPA were equal or significantly higher than RIs of the nucleoli in the same cells. A comparison with the low-density condensates, viz., speckles and stress granules (15), is perhaps even more interesting. These have been defined and studied exhaustively by means of fluorescence imaging but do not present any measurable signal in the RI. At the same time, it was shown that they remain permeable to diffusing fluorescence protein probes, indicating an open, porous structure. This is consistent, then, with the notion of a minimal protein-protein connectivity required for the dielectric polarizability to reflect a material continuum. Thus,

the condensed state reported by RI is subtly different from the notion of thermodynamic phase separation per se. As had been pointed out (15), phase separation may result, for example, from depletion interaction or polymer segregation, rather than associative interaction, or from sparse and possibly labile cross-links between long polymers such as RNA. Such low-density condensates may appear in fluorescence without forming a polarizable dielectric continuum distinct from the solvent.

The simple two-phase coexistence model for phase separation posits a uniform concentration within each of the high- and low-density regions. Clearly, in the example of Fig. 7, the condensed phase is not uniform, and a certain phase separation occurs within. This complicates the simple picture of two-phase equilibrium but may offer an experimental paradigm for layering or compartmentalization of more complex mixtures. Such multicomponent systems have been addressed theoretically (33) and recall the core-shell structure reported for nucleoli on the basis of fluorescence observations (34). Although it was not the aim of the present study, we may also point out that the RI contrast of nucleoli suggests some internal compartmentalization.

CONCLUSION

In summary, we show that RI mapping is a useful addition to the tool chest for study of protein condensation and liquid-liquid phase separation. It is especially useful for study of live cells where photodamage is a major concern. In comparison with fluorescence imaging, it is quantifiable and much less subject to artifacts such as saturation (high or low) or effects of out-of-focus sources. Combination of 3D RI mapping with high-dynamic-range confocal imaging would offer a further step forward for analysis. Using synthetic ferritin-based condensates expressed in living cells, we have shown that the RI reveals a transformation from an elevated local concentration to a truly condensed material phase with an elevated optical polarizability. This should provide further clarification and classification of biomolecular condensates and their condensation dynamics.

ACKNOWLEDGMENTS

The authors acknowledge the lab of Cliff Brangwynne for provision of Corelet plasmid sources, with special thanks to Dan Bracha for discussions and comments. We acknowledge the de Picciotto Cancer Cell Observatory, In Memory of Wolfgang and Ruth Lesser, in which the NanoLive 3D CellExplorer microscope was operated, and especially the assistance of Joseph Addadi for guidance in its use. This work was funded in part by the US-Israel Binational Science Foundation and by the European Union, ERC AdG, CryoSTEM, 101055413. (Views and opinions expressed are however those of the authors only and do not necessarily reflect those of the European Union or the European Research Council. Neither the European Union nor the granting authority can be held responsible for them.) M.E. is incumbent of the Sam and Ayala Zacks Professorial Chair in Chemistry. The Elbaum lab has benefited from the historical generosity of the Harold Perlman family.

AUTHOR CONTRIBUTIONS

O.M.: investigation, data curation, visualization, and writing – original draft; P.K.: validation, data curation, visualization, writing – original draft, and writing – review & editing; A.D.: investigation and resources; Y.B.: resources; M.E.: conceptualization, methodology, validation, supervision, project administration, funding acquisition, writing – original draft, and writing – review & editing.

DECLARATION OF INTERESTS

The authors declare no competing interests.

SUPPORTING MATERIAL

Supporting material can be found online at https://doi.org/10.1016/j.bpr.2025.100235.

REFERENCES

1. Banani, S. F., H. O. Lee, …, M. K. Rosen. 2017. Biomolecular condensates: organizers of cellular biochemistry. *Nat. Rev. Mol. Cell Biol.* 18:285–298. https://doi.org/10.1038/nrm.2017.7.
2. Brangwynne, C. P., C. R. Eckmann, …, A. A. Hyman. 2009. Germline P Granules Are Liquid Droplets That Localize by Controlled Dissolution/Condensation. *Science.* 324:1729–1732. https://doi.org/10.1126/science.1172046.
3. Garcia-Seisdedos, H., C. Empereur-Mot, …, E. D. Levy. 2017. Proteins evolve on the edge of supramolecular self-assembly. *Nature.* 548:244–247. https://doi.org/10.1038/nature23320.
4. Schweke, H., M. Pacesa, …, E. D. Levy. 2024. An atlas of protein homo-oligomerization across domains of life. *Cell.* 187:999–1010.e15. https://doi.org/10.1016/j.cell.2024.01.022.
5. Sawaya, M. R., M. P. Hughes, …, D. S. Eisenberg. 2021. The expanding amyloid family: Structure, stability, function, and pathogenesis. *Cell.* 184:4857–4873. https://doi.org/10.1016/j.cell.2021.08.013.
6. Mukherjee, S., M. Poudyal, …, S. K. Maji. 2024. Protein misfolding and amyloid nucleation through liquid–liquid phase separation. *Chem. Soc. Rev.* 53:4976–5013. https://doi.org/10.1039/D3CS01065A.
7. Elbaum-Garfinkle, S. 2019. Matter over mind: Liquid phase separation and neurodegeneration. *J. Biol. Chem.* 294:7160–7168. https://doi.org/10.1074/jbc.REV118.001188.
8. Heidenreich, M., J. M. Georgeson, …, E. D. Levy. 2020. Designer protein assemblies with tunable phase diagrams in living cells. *Nat. Chem. Biol.* 16:939–945. https://doi.org/10.1038/s41589-020-0576-z.
9. Bellapadrona, G., and M. Elbaum. 2014. Supramolecular Protein Assemblies in the Nucleus of Human Cells. *Angew. Chem. Int. Ed.* 53:1534–1537. https://doi.org/10.1002/anie.201309163.
10. Bellapadrona, G., S. Sinkar, …, M. Elbaum. 2015. Supramolecular Assembly and Coalescence of Ferritin Cages Driven by Designed Protein–Protein Interactions. *Biomacromolecules.* 16:2006–2011. https://doi.org/10.1021/acs.biomac.5b00435.

11. Bellapadrona, G., and M. Elbaum. 2016. Design of a Redox-Sensitive Supramolecular Protein Assembly System Operating in Live Cells. *Nano Lett.* 16:6231–6235. https://doi.org/10.1021/acs.nanolett.6b02480.

12. Bracha, D., M. T. Walls, …, C. P. Brangwynne. 2018. Mapping Local and Global Liquid Phase Behavior in Living Cells Using Photo-Oligomerizable Seeds. *Cell.* 175:1467–1480.e13. https://doi.org/10.1016/j.cell.2018.10.048.

13. McCall, P. M., K. Kim, …, J. Brugués. 2023. Label-free composition determination for biomolecular condensates with an arbitrarily large number of components. https://doi.org/10.1101/2020.10.25.352823.

14. Kim, K., S. Lee, …, Y. Park. 2016. Three-dimensional label-free imaging and quantification of lipid droplets in live hepatocytes. *Sci. Rep.* 6:36815. https://doi.org/10.1038/srep36815.

15. Kim, T., J. Yoo, …, Y. Shin. 2023. RNA-mediated demixing transition of low-density condensates. *Nat. Commun.* 14:2425. https://doi.org/10.1038/s41467-023-38118-z.

16. Nygate, Y. N., M. Levi, …, N. T. Shaked. 2020. Holographic virtual staining of individual biological cells. *Proc. Natl. Acad. Sci. USA.* 117:9223–9231. https://doi.org/10.1073/pnas.1919569117.

17. Park, Y., C. Depeursinge, and G. Popescu. 2018. Quantitative phase imaging in biomedicine. *Nat. Photonics.* 12:578–589. https://doi.org/10.1038/s41566-018-0253-x.

18. Schürmann, M., J. Scholze, …, C. J. Chan. 2016. Cell nuclei have lower refractive index and mass density than cytoplasm. *J. Biophot.* 9:1068–1076. https://doi.org/10.1002/jbio.201500273.

19. Kim, G., H. Hugonnet, …, Y. Park. 2024. Holotomography. *Nat. Rev. Methods Primers.* 4:51. https://doi.org/10.1038/s43586-024-00327-1.

20. Irgen-Gioro, S., S. Yoshida, …, S. Chong. 2022. Fixation can change the appearance of phase separation in living cells. *eLife.* 11:e79903. https://doi.org/10.7554/eLife.79903.

21. Schindelin, J., I. Arganda-Carreras, …, A. Cardona. 2012. Fiji: an open-source platform for biological-image analysis. *Nat. Methods.* 9:676–682. https://doi.org/10.1038/nmeth.2019.

22. Shin, Y., Y.-C. Chang, …, C. P. Brangwynne. 2018. Liquid Nuclear Condensates Mechanically Sense and Restructure the Genome. *Cell.* 175:1481–1491.e13. https://doi.org/10.1016/j.cell.2018.10.057.

23. Xia, J., J. Z. Zhao, …, C. P. Brangwynne. 2025. Chromatin heterogeneity modulates nuclear condensate dynamics and phase behavior. *Nat. Commun.* 16:6406. https://doi.org/10.1038/s41467-025-60771-9.

24. Patel, A., H. O. Lee, …, S. Alberti. 2015. A Liquid-to-Solid Phase Transition of the ALS Protein FUS Accelerated by Disease Mutation. *Cell.* 162:1066–1077. https://doi.org/10.1016/j.cell.2015.07.047.

25. Shin, Y., J. Berry, …, C. P. Brangwynne. 2017. Spatiotemporal Control of Intracellular Phase Transitions Using Light-Activated optoDroplets. *Cell.* 168:159–171.e14. https://doi.org/10.1016/j.cell.2016.11.054.

26. Biswas, A., O. Muñoz, …, S. Reber. 2025. Conserved nucleocytoplasmic density homeostasis drives cellular organization across eukaryotes. *Nat. Commun.* 16:7597. https://doi.org/10.1038/s41467-025-62605-0.

27. Strom, A. R., Y. Kim, …, C. P. Brangwynne. 2024. Condensate interfacial forces reposition DNA loci and probe chromatin viscoelasticity. *Cell.* 187:5282–5297.e20. https://doi.org/10.1016/j.cell.2024.07.034.

28. Beck, T., L.-M. van der Linden, …, J. Guck. 2024. Optical characterization of molecular interaction strength in protein condensates. *MBoC.* 35:ar154. https://doi.org/10.1091/mbc.E24-03-0128.

29. Kassimi, N. E.-B., and A. J. Thakkar. 2009. A simple additive model for polarizabilities: Application to amino acids. *Chem. Phys. Lett.* 472:232–236. https://doi.org/10.1016/j.cplett.2009.03.016.

30. Zhao, H., P. H. Brown, and P. Schuck. 2011. On the Distribution of Protein Refractive Index Increments. *Biophys. J.* 100:2309–2317. https://doi.org/10.1016/j.bpj.2011.03.004.

31. Khago, D., J. C. Bierma, …, R. W. Martin. 2018. Protein refractive index increment is determined by conformation as well as composition. *J. Phys. Condens. Matter.* 30:435101. https://doi.org/10.1088/1361-648X/aae000.

32. Möckel, C., T. Beck, …, J. Guck. 2024. Estimation of the mass density of biological matter from refractive index measurements. *Biophys. Rep.* 4:100156. https://doi.org/10.1016/j.bpr.2024.100156.

33. Jacobs, W. M., and D. Frenkel. 2017. Phase Transitions in Biological Systems with Many Components. *Biophys. J.* 112:683–691. https://doi.org/10.1016/j.bpj.2016.10.043.

34. Brangwynne, C. P., T. J. Mitchison, and A. A. Hyman. 2011. Active liquid-like behavior of nucleoli determines their size and shape in Xenopus laevis oocytes. *Proc. Natl. Acad. Sci. USA.* 108:4334–4339. https://doi.org/10.1073/pnas.1017150108.

Biophysical Reports, Volume *5*

Supplemental information

Refractive index as an indicator for dynamic protein condensation in cell nuclei

Orlando Marin, Peter Kirchweger, Arina Dalaloyan, Yoav Barak, and Michael Elbaum

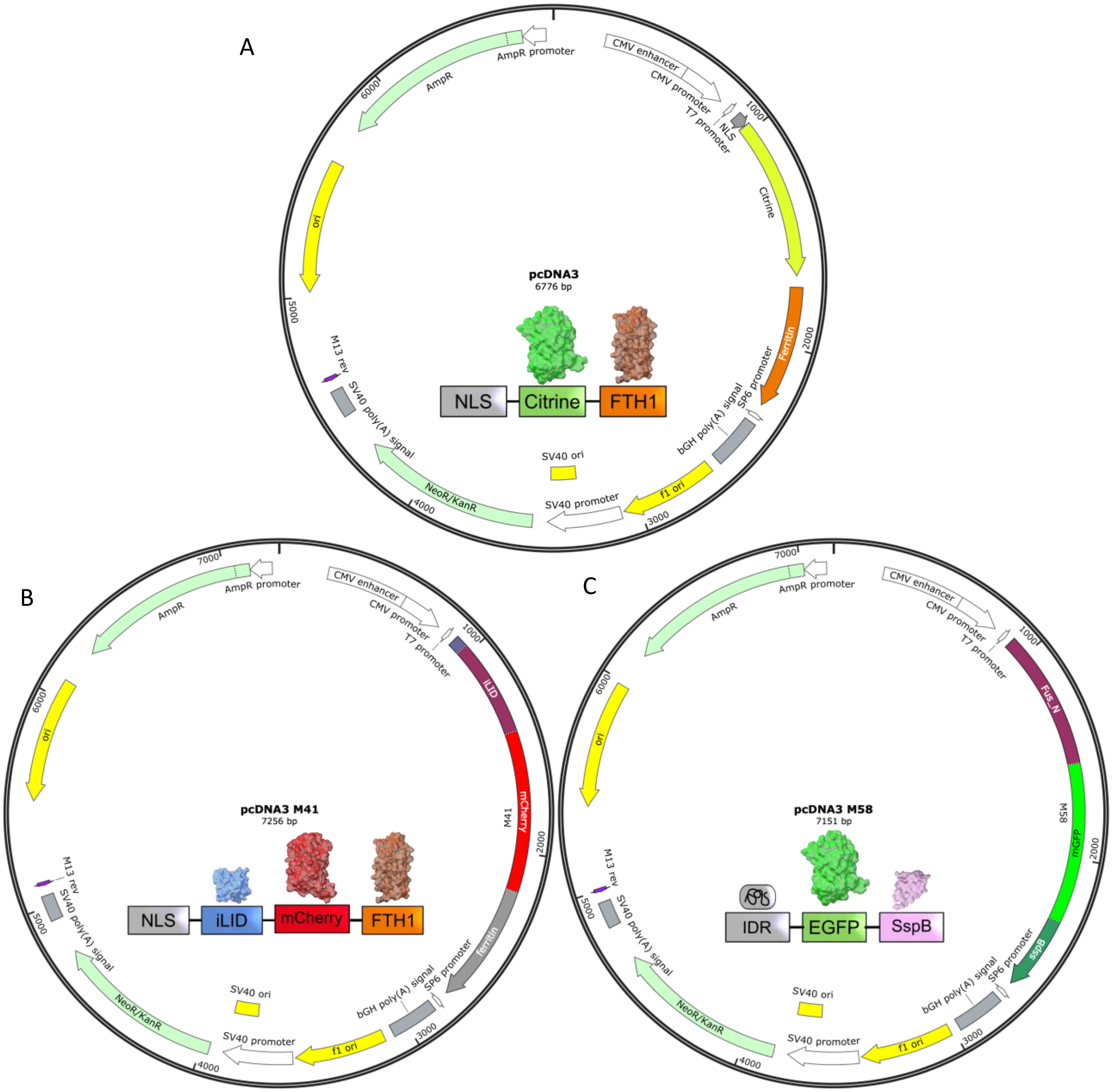

Figure S1: Plasmid maps of (A) SMPA, (B) the first and (C) second part of the Correlet system in the pcDNA3 vector: (A) NLS-Citrine-ferritin, (B) NLS-iLid-mCherry-Ferritin, and (C) IDR region of FUS_N-EGFP-SspB.

PBD IDs used in Fig. 1: **GFP**: 1GFL: https://doi.org/10.2210/pdb1GFL/pdb; **24-mer human ferritin**: 5N27: https://doi.org/10.2210/pdb5N27/pdb; **iLID domain**: 4WF0: https://doi.org/10.2210/pdb4WF0/pdb; **mCHerry**: 6YLM: https://doi.org/10.2210/pdb6YLM/pdb; **SspB**: 1OX9: https://doi.org/10.2210/pdb1OX9/pdb.

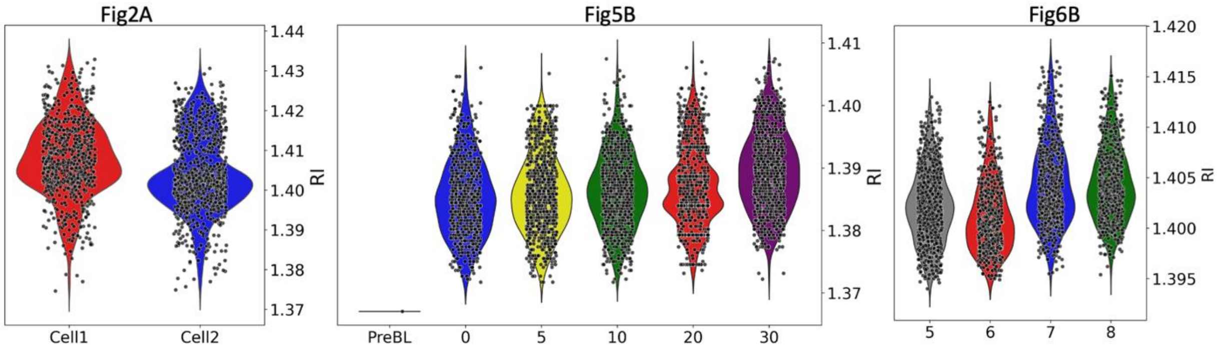

Figure S2: Violin plots showing the individual data points used to produce the plots. The left, middle and right panel show the plots from Fig 2A, Fig5B, and from Fig6B, respectively.

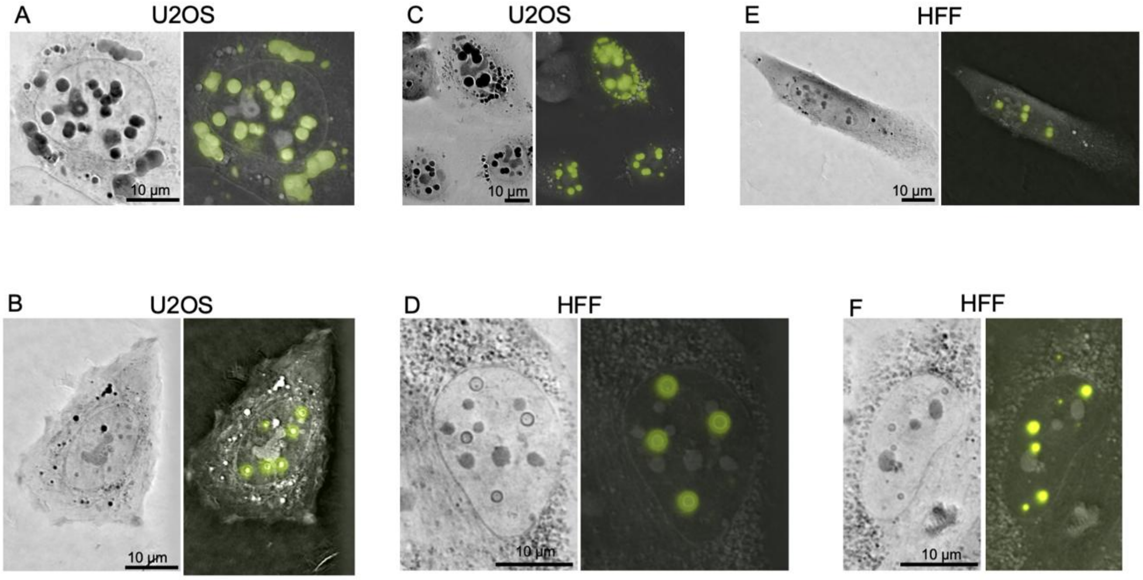

Figure S3. Additional Examples of SMPA in U2OS and HFF cells. SMPA can be reproduced in U2OS (A,B,C) and HFF cells (D,E,F). The thin spherical shells are seen in both cell types (B, D, F). Scale bars are 10 µm.

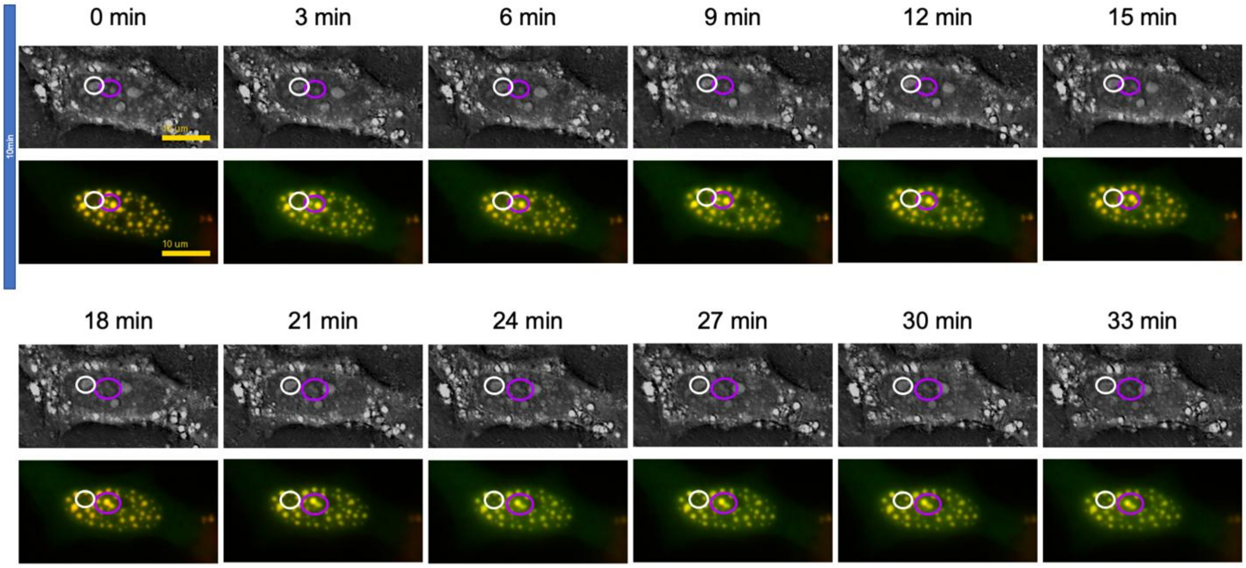

Figure S4: Long observation of Corelets. After 10 min blue light exposure, the same cell was imaged for 33 min (1x per minute); an image every 3 min is depicted. The purple outline indicates a persistent Corelet, and the white outline is a non-fluorescent nucleolus.

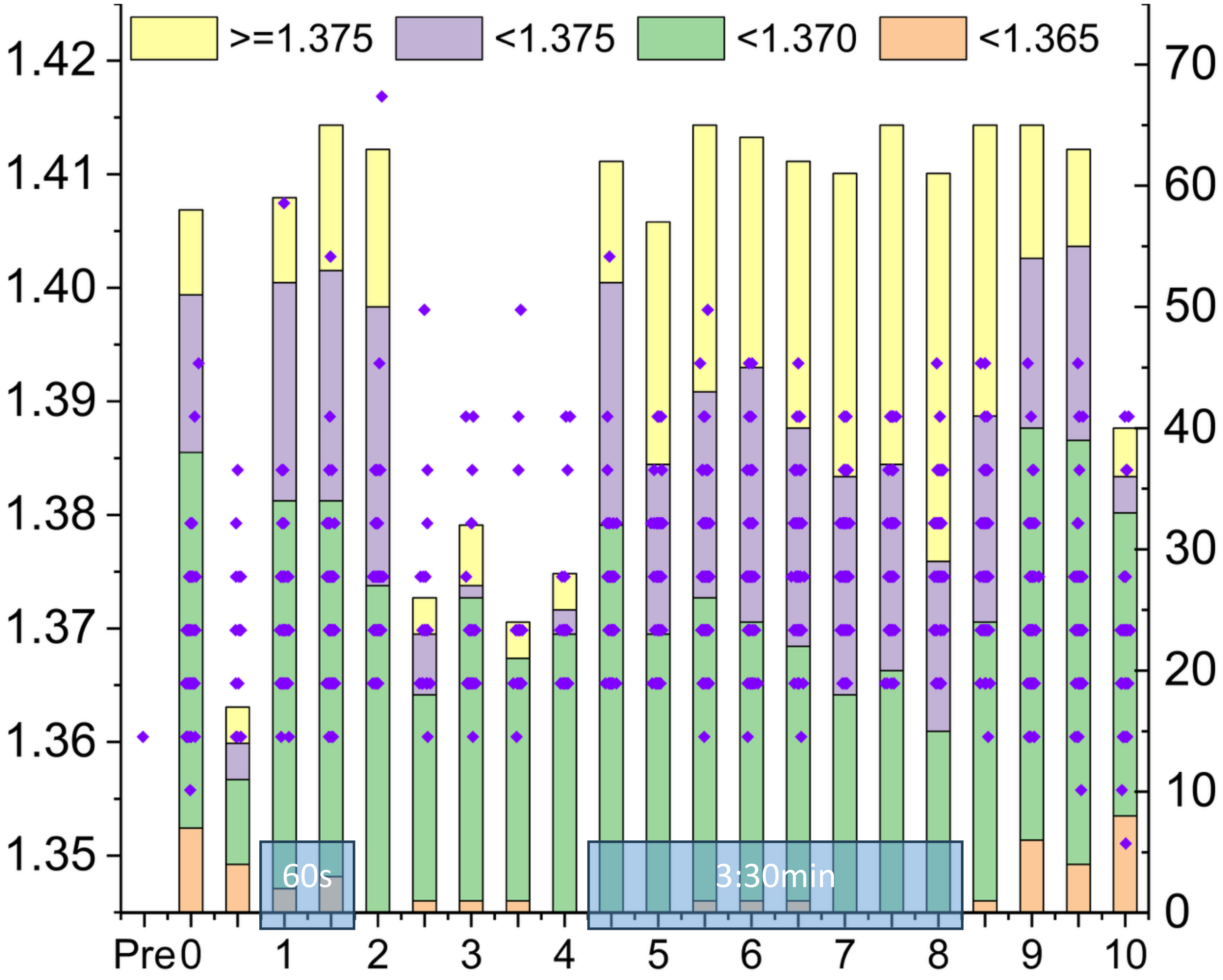

Figure S5: Quantification of Maximum values from Fig8. Plotted are rounded MAX values of the ROIs extracted from the timeseries shown in Fig8. Purple shows the actual datapoints, and counts appear on the right side vertical axis. Orange indicates MAX values below 1.365, green between 1.365 and 1.37, purple between 1.37 and 1.375, and yellow shows values above 1.375. The number of median and maximum values in each category are shown in Table S1.

TIME	TOTAL DATAPOINTS		<1.365		<1.370		<1.375		>=1.375	
	MED	MAX	MED	MAX	MED	MAX	MED	MAX	MED	MAX
**0**	54	58	33	7	21	31	0	13	0	7
**0.5**	15	17	9	4	5	7	1	3	0	3
**1**	54	59	27	2	26	32	1	18	0	7
**1.5**	54	65	25	3	26	31	3	19	0	12
**2**	58	63	26	0	30	27	1	23	1	13
**2.5**	25	26	14	1	11	17	0	5	0	3
**3**	29	32	19	1	10	25	0	1	0	5
**3.5**	24	24	14	1	10	20	0	0	0	3
**4**	29	28	17	0	11	23	1	2	0	3
**4.5**	57	62	25	0	31	32	0	20	1	10
**5**	56	57	28	0	28	23	0	14	0	20
**5.5**	60	65	34	1	26	25	0	17	0	22
**6**	64	64	32	1	32	23	0	21	0	19
**6.5**	62	62	30	1	32	21	0	18	0	22
**7**	59	61	27	0	32	18	0	18	0	25
**7.5**	60	65	23	0	35	20	2	17	0	28
**8**	58	61	25	0	33	15	0	14	0	32
**8.5**	63	65	23	1	38	23	2	17	0	24
**9**	56	65	28	6	27	34	1	14	0	11
**9.5**	56	63	28	4	27	35	1	16	0	8
**10**	32	40	21	8	11	25	0	3	0	4

Table S1: Analysis of Fig 8. Number of Datapoints in the categories below 1.365, between 1.365 and 1.37, between 1.37 and 1.375, and greater than 1.375.

Movie captions:

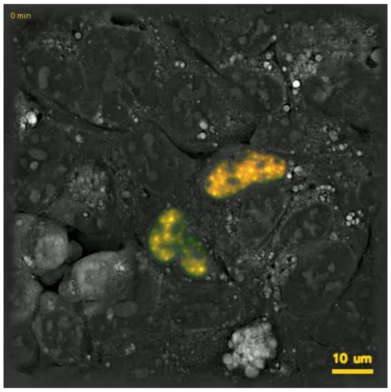

Movie S1: Image compilation the time series depicted in Figure 4. The entire field of view is shown as an overlay of the three channels. Scale bar 10 µm. Time stamps appear at the upper left corner.

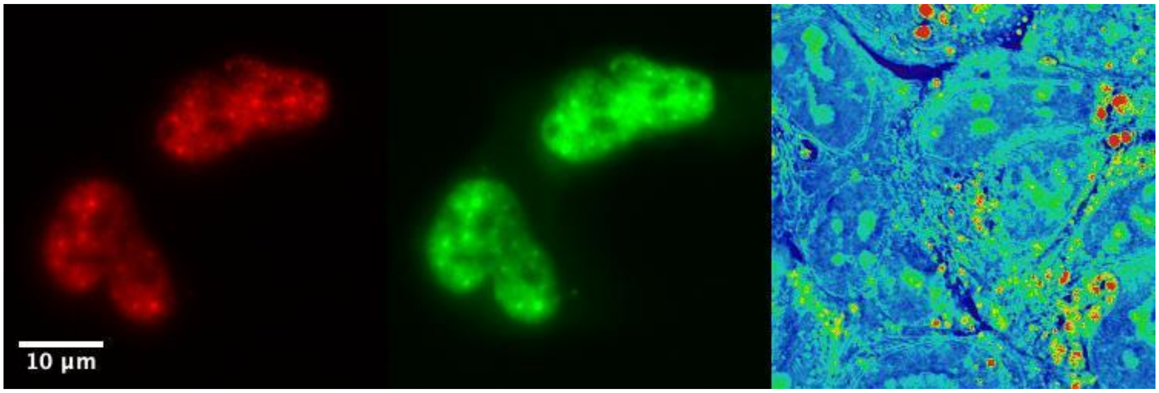

Movie S2: Image compilation of the Ft-mCherry, FUS-GFP and RI channels of the time series depicted in Figure 4. Blue frames indicate the timing of the photo-activation. The same area is shown as in Fig 4. Scale bar 10 µm.

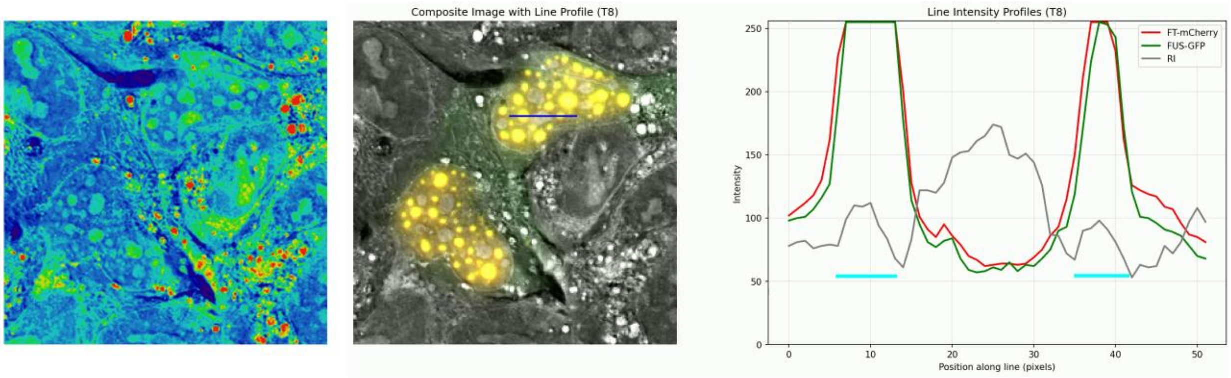

Movie S3: A line profile across the time series of Figure 4. The panel on the left repeats the frames in Fig 4. The center panel shows a composite image with combined fluorescence overlaid. The frame on the right shows intensity traces across the line shown in blue in the center panel. The green, red and black lines represent the FUS-GFP, Ft-mCherry and RI channels, respectively. The elevated RI between the two fluorescence peaks is a nucleolus. Note the dynamic response of the RI underneath the fluorescence peaks. (Vertical scales are shown with arbitrary units on an 8-bit scale.) Horizontal blue bars on the graph indicate that the measurement was immediately preceded by 30 sec photo-activation, and their placement indicates the intersection of the analysis line with fluorescent foci.

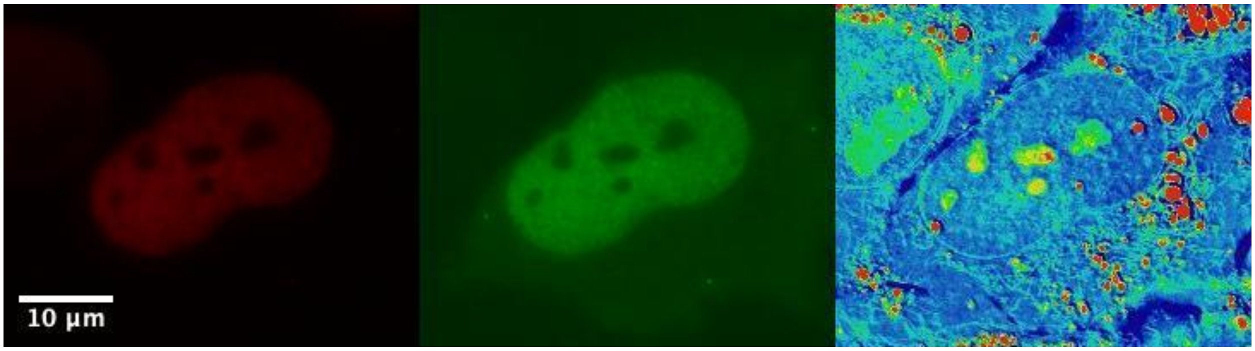

Movie S4: Image compilation of the Ft-mCherry, FUS-GFP, and the RI time series depicted in Figure 8. Images were recorded every 30 seconds. Saturated fluorescence frames indicate the periods of photo-activation, corresponding to the blue bars shown in the figure.
